# A rice calcium-dependent protein kinase is expressed in cortical root cells during the presymbiotic phase of the arbuscular mycorrhizal symbiosis

**DOI:** 10.1186/1471-2229-11-90

**Published:** 2011-05-19

**Authors:** Lidia Campos-Soriano, Jorge Gómez-Ariza, Paola Bonfante, Blanca San Segundo

**Affiliations:** 1 Centre for Research in Agricultural Genomics (CRAG) CSIC-IRTA-UAB. Department of Molecular Genetics. Campus UAB, Edifici CRAG, Bellaterra (Cerdanyola del Vallès) 08193 Barcelona, Spain; 2 Department of Plant Biology, University of Torino and Istituto per la Protezione delle Piante - CNR. Sezione di Torino. Viale P.A. Mattioli 25, Torino 10125, Italy

## Abstract

**Background:**

The arbuscular mycorrhizal (AM) symbiosis consists of a mutualistic relationship between soil fungi and roots of most plant species. This association provides the arbuscular mycorrhizal fungus with sugars while the fungus improves the uptake of water and mineral nutrients in the host plant. Then, the establishment of the arbuscular mycorrhizal (AM) symbiosis requires the fine tuning of host gene expression for recognition and accommodation of the fungal symbiont. In plants, calcium plays a key role as second messenger during developmental processes and responses to environmental stimuli. Even though calcium transients are known to occur in host cells during the AM symbiosis, the decoding of the calcium signal and the molecular events downstream are only poorly understood.

**Results:**

The expression of seventeen Calcium-dependent Protein Kinase (CPK) genes representative of the four distinct phylogenetic groups of rice CPKs was monitored during the presymbiotic phase of the AM symbiosis. Among them, *OsCPK18 *and *OsCPK4*, were found to be transcriptionally activated in response to inoculation with the AM fungus *Glomus intraradices. OsCPK18 *and *OsCPK4 *gene expression was also up-regulated by fungal-produced diffusible molecules. Laser microdissection revealed expression of *OsCPK18 *in cortical cells, and not in epidermal cells of *G. intraradices*-inoculated rice roots, suggesting a preferential role of this gene in the root cortex. Moreover, a plasma membrane localization of OsCPK18 was observed by transient expression assays of green fluorescent protein-tagged OsCPK18 in onion epidermal cells. We also show that the myristoylation site of the OsCPK18 N-terminus is required for plasma membrane targeting.

**Conclusion:**

The rapid activation of *OsCPK18 *expression in response to AM inoculation, its expression being also induced by fungal-secreted signals, together with the observed plasma membrane localization of OsCPK18, points to a role for OsCPK18 in perception of the AM fungus. The *OsCPK18 *gene might be considered as a marker for the presymbiotic phase of the symbiotic process. These findings provide a better understanding of the signaling mechanisms operating during the AM symbiosis and will greatly facilitate their molecular dissection.

## Background

Most vascular flowering plants have the ability to establish symbiotic associations with arbuscular mycorrhizal (AM) fungi [1]. The main benefit for the plant is improved uptake of water and mineral nutrients from the soil, particularly phosphate, in exchange for photosynthetically fixed carbon [2]. The mycorrhizal symbiosis has been also associated with increased resistance to pathogen infection and tolerance to abiotic stress in several plant species [3]. As a consequence, the AM symbiosis is of tremendous significance in agricultural ecosystems.

The legumes *Medicago truncatula *and *Lotus japonicus *have been widely adopted as the reference species for studies of the AM symbiosis. Contrary to this, *Arabidopsis thaliana*, the model system for functional genomics in plants, has no mycorrhization ability. Rice, a monocotyledonous plant with a completely sequenced genome, establishes symbiotic associations with mycorrhizal fungi [4,5]. As compared to the model legume species, the genes responsible for the AM symbiotic interaction in rice are less characterized.

Successful symbiosis with AM fungi relies on the fine tuning and appropriate control of host gene expression and physiological responses. A molecular dialogue is early established between the host plant and the AM fungus and prepares the two partners for the subsequent root colonization. Signal exchange and communication starts prior to the initial cell-to-cell contact between the symbionts. Thus, plant roots exude strigolactones which have an stimulatory effect on AM growth [6]. Fungal hyphae, in turn, produce diffusible molecules, the "Myc factors" (analogous to the rhizobial Nod factors). Very recently, it was reported that the AM fungus secretes lipochitooligosaccharides which stimulate formation of AM symbiosis in diverse plant species [7]. Perception of Myc factors by the host cells triggers a rapid and transient elevation of intracellular calcium, alterations in the cellular architecture and transcriptional reprogramming of the root [8-12]. Even though both cytoplasmic [10] and nuclear [9] pre-infection Ca^2+ ^spiking responses are elicited in *M. truncatula *roots in response to AM fungi, the mechanisms by which Ca^2+ ^alterations are sensed and transduced into early AM-induced signaling remain unknown.

Once contact between the symbionts is established, the fungus enters into the root through the epidermal cells, and penetrates into the cortex where it forms highly branched structures, called arbuscules, in the cortical cells of the root. The arbuscules are the site of the major nutrient exchange between the two symbionts [2,13,14].

It is also known that the plant response to Myc factors is mediated by a partially characterized signaling pathway which is required for the establishment of both rhizobial and AM symbioses, the so called common symbiosis (SYM) pathway [2,13-15]. Forward genetic analysis in the model legumes *Medicago truncatula *and *Lotus japonicus *has led to the identification of components of the SYM signaling pathway. They are: a leucine-rich-repeat receptor-like kinase, the *SYMRK *protein in *L. japonicus *(known as *DMI2 *for "Does Not Make Infections 2" in *M. truncatula*), two nucleoporins (*NUP85 *and *NUP133*), two cation channel proteins (the *L. japonicus CASTOR *and *POLLUX *proteins; *DMI1a *and *DMI1b *in *M. truncatula*), a calcium and calmodulin-dependent protein kinase (*CCaMK *in *L. japonicus*; *DMI3 *in *M. truncatula*) and CYCLOPS (*LjCYCLOPS*; DIM3-interacting protein in *M. truncatula*) [16-21]. CCaMK interacts with, and phosphorylates, CYCLOPS in the nucleus [21,22]. In rice, the function of several SYM genes appears to be conserved, including *CASTOR *and *POLLUX *(acting upstream of the calcium-spiking signal) and *CCaMK *and CYCLOPS (acting downstream of the calcium-spiking signal) [23-25]. Evidence also support the existence of alternative, SYM-independent signaling pathways controlling the early responses to AM fungi in both rice and *M. truncatula *[25,26].

Transcript profiling of mycorrhizal roots allowed the identification of AM-regulated genes in several plant species, including rice [3,27-30]. However, the majority of these studies focused on the mature phase of the symbiotic process, a period in which the host root is already colonized and arbuscules are developed in the root cortical cells. Along with this, alterations in the expression of genes connected to nutrient acquisition processes, such as phosphate transporter genes, are well documented in different AM associations [31,32]. Genes involved in cellular modifications, transcriptional control and defense-related responses are also known to be regulated during the AM symbiosis [4,31].

Even though alterations in Ca^2+ ^levels are known to occur in host cells during the presymbiotic phase, the decoding of the calcium signal is only poorly understood. On the other hand, it is well established that Calcium-dependent protein kinases (CPKs or CDPKs) are important Ca^2+ ^sensors in signaling processes during growth, development and stress responses in plants [33,34]. CPKs belong to the CDPK/SnRK superfamily of protein kinases and represent a differentiated group of protein kinases found in plants, algae and protists [34-36]. They possess a characteristic structure consisting of four domains: an amino terminal variable domain, a serine/threonine kinase domain, a junction autoinhibitory domain, and a C-terminal calmodulin domain. These features make CPKs ideally structured to rapidly perceive alterations in intracellular calcium concentration and translating them into protein phosphorylation cascades. CPK functioning is, however, different from that of CCaMK functioning, since CPKs do not require calmodulin for their activation. Ca^2+ ^binds directly the calmodulin domain of CPKs and induces a conformational change resulting in kinase activation [34]. The available information on plant CPKs from various plant species indicates that they are encoded by multigene families and that whereas some of the genes are ubiquitously expressed, others show a tissue-specific pattern of expression or are regulated by stress (wounding, salinity, cold, drought, pathogen infection) [33,37,38].

Knowing that Ca^2+ ^plays a central role in the AM-induced signaling pathway, it was of interest to investigate to what extent CPKs are involved in the AM-induced signaling pathway. Towards this goal, the expression pattern of seventeen *cpk *genes was monitored in rice plants that have been inoculated with the AM fungus *Glomus intraradices*. We provide evidence that the expression of two distinct *cpk *genes, the *OsCPK18 *and *OsCPK4 *genes, is rapidly induced during the presymbiotic phase of the rice/*G. intraradices *interaction. *OsCPK18 *and *OsCPK4 *gene expression is also activated by fungal-produced diffusible fungal signal(s). By using the laser microdissection (LMD) technology, *OsCPK18 *expression was detected in cortical cells, but not epidermal cells, of the *G. intraradices*-inoculated rice roots. Moreover, a plasma membrane localization of OsCPK18 is here reported, the myristoylation site of OsCPK18 being required for its plasma membrane localization. Together, these findings support that OsCPK18 might play a role during recognition of the AM fungus by the host cells.

## Results

### Expression of *CPK *genes in AM-inoculated rice roots

A genome-wide analysis of rice *CPK *genes identified 31 genes which are distributed into four phylogenetic groups (I-IV) [39,40]. Moreover, a comparison of the rice *CPK *genes distinguished 11 closely related pairs which, most probably, have arisen via sequential duplication events, the *OsCPK1/15*, *OsCPK2/14, OsCPK3/16, OsCPK4/18, OsCPK5/13, OsCPK7/23, OsCPK8/20, OsCPK11/17, OsCPK21/22, OsCPK24/28 *and *OsCPK25/26 *pairs [40]. Based of the homology and phylogenetic relatedness among the rice *CPK *genes, we selected a subset of seventeen *CPK *genes representative of the four distinct phylogenetic groups of rice CPKs in which at least one representative member for each pair of closely related *CPK *genes was present. The subset of genes assayed in this work included *OsCPK7, OsCPK10, OsCPK13*, *OsCPK17 *and *OsCPK24 *from Group I; *OsCPK2*, *OsCPK15*, *OsCPK19 *and *OsCPK25 *from Group II; *OsCPK8, OsCPK9*, *OsCPK16 *and *OsCPK22 *from Group III; and *OsCPK4*, *OsCPK18*, *OsCPK*30 and *OsCPK31 *from Group IV.

The expression pattern of selected rice *CPK *genes and their transcriptional response to inoculation with the AM fungus *G. intraradices*, were examined during the presymbiotic phase of the symbiotic process. A preliminary screening was carried out by semiquantitative RT-PCR experiments with RNA samples obtained from whole rice roots that had been inoculated with fungal spores using the single sandwich method. Total RNA was isolated at 24, 48, 72 and 96 hours after inoculation of the rice roots with *G. intraradices*, as well as from mock-inoculated rice roots. Many *CPK *genes were found to be expressed in rice roots and at different levels (Additional file 1: Figure S1). Among them, the *OsCPK4 *and *OsCPK18 *genes were expressed at the highest levels. Moderate to low levels of expression were observed for *OsCPK10*, *OsCPK13*, *OsCPK17*, *OsCPK24*, *OsCPK15*, *OsCPK19*, *OsCPK8 *and *OsCPK9*, whereas *OsCPK30 *transcripts were barely detected (Additional file 1: Figure S1). The *OsCPK7 *and *OsCPK16 *genes showed expression profiles similar to those shown for *OsCPK8 *and *OsCPK30*, respectively (results not shown). Taken in the whole, the expression level of the various *CPK *genes here investigated appears not to be dramatically affected upon inoculation with *G. intraradices*, with the exception of *OsCPK4 *and *OsCPK18 *expression (Additional file 1: Figure S1)

For a comparison, the expression of the known *SYM *genes from rice, namely the *OsSYMRK*, *OsPOLLUX*, *OsCASTOR *and *OsCCaMK *genes, was also examined. This analysis revealed up-regulation of *OsSYMRK*, *OsPOLLUX *and *OsCCaMK *in response to *G. intraradices *at 72 and 96 hours post-inoculation (Additional file 1: Figure S1). The observed induction of these genes indicates that the host plant cells perceive and respond to the AM fungus through the activation of the AM-specific SYM signaling pathway.

**Figure 1:**
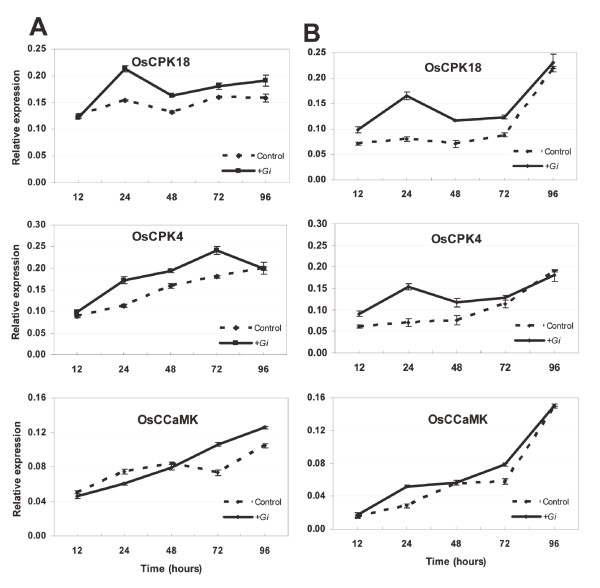
**Gene expression analysis by real-time qPCR for the *OsCPK18*, *OsCPK4 *and *OsCCaMK *genes**. The single-sandwich (A) or the double-sandwich (B) system was used for inoculation. Roots inoculated with *G. intraradices *and mock-inoculated roots were harvested at the indicated times. Each sample consisted on a pool of at least 12 individual plants. Expression levels are shown relative to the housekeeping *OsAct1 *gene. Data shown represents the means ± error. Three independent experiments were carried out with similar results.

Since RT-PCR analyses do not provide reliable quantitative data of gene expression, quantitative reverse transcription-PCR (RT-qPCR) was used to further characterize the effect of *G. intraradices *inoculation on *OsCPK4 *and *OsCPK18 *gene expression. By using the single sandwich system for fungal inoculation, up-regulation of *OsCPK18 *gene expression occurred as early as 24 h post-inoculation with *G. intraradices *(Figure 1A, upper panel). The level of *OsCPK18 *transcripts remained higher at the subsequent time points in the *G. intraradices*-inoculated roots compared to mock-inoculated roots. Concerning *OsCPK4*, its expression was also found to be up-regulated in response to *G. intraradices *inoculation during the time period of 24-72 hours. *OsCPK4 *expression returned to a level similar to that of non-inoculated roots by 96 h post-inoculation (Figure 1A, middle panel). Finally, *OsCCaMK *expression increased in *G. intraradices*-infected roots relative to mock-inoculated roots by 72-96 hours post-inoculation (Figure 1A, lower panel).

Overall, gene expression studies revealed up-regulation of the rice *OsCPK4 *and *OsCPK18 *genes during the presymbiotic phase of the AM symbiosis. These results were consistently observed in all three independent experiments. Although the expression of the *OsCPK18, OsCPK4 *and *OsCCaMK *genes was up-regulated in AM-inoculated roots compared to non-inoculated roots, it is also true that the amplitude of the differential expression for these genes was not very high during the time period here analyzed. Concerning *OsCCaMK *for which a role during AM symbiosis has been demonstrated in rice [23], its variation in the expression level in response to AM inoculation is also low and appears to occur at a later time point compared to the observed activation of Os*CPK18 *and *OsCPK4 *gene expression.

### Diffusible factors released by *G. intraradices *induce *OsCPK18 *and *OsCPK4 *expression in rice roots

It is generally assumed that plants perceive AM fungi even before physical contact between the two symbionts, and that recognition of Myc factors triggers alterations in Ca^2+ ^levels and transcriptional responses in host roots [7,9,11,41]. In this work, the double sandwich method was used to investigate whether the observed induction of *OsCPK18 *and *OsCPK4 *expression is attributable to diffusible factors released by the fungus. This system prevents contact between the two symbionts while allowing the exchange of signal molecules [42]. *OsCPK18 *and *OsCPK4 *expression was analyzed by RT-qPCR (Figure 1B). When using the double sandwich system for inoculation of rice roots, *OsCPK18 *and *OsCPK4 *expression was found to be rapidly activated in response to *G. intraradices *inoculation (Figure 1B, upper and medium panel). However, induction of *OsCPK4 *and *OsCPK18 *expression was not maintained with time (the maximum induction occurred at 24 h post-inoculation for the two genes). Similar levels of transcript accumulation were observed in *G. intraradices*- and mock-inoculated roots at the latest time point here analyzed (96 hours post-inoculation). From these results it can be concluded that a diffusible fungal factor elicits expression of the rice *OsCPK18 *and *OsCPK4 *genes, and that this activation is transient. Most probably, contact between the two partners is needed to maintain the expression of these genes in an activated manner at the subsequent stages of the infection process. Under the same experimental conditions, an activation of *OsCCaMK *gene expression also occurred at 24 h post-inoculation although differences in *OsCCaMK *gene expression between AM-inoculated and mock inoculated roots were lower than those observed for the *CPK *genes (Figure 1B, lower panel).

### *OsCPK18 *expression in microdissected root cells

The laser-microdissection (LMD) technology has been successfully used for gene expression analysis in arbuscule-containing cells in different plant species such as *Medicago*, *L*o*tus *or tomato [27-29,43,44]. A variety of protocols have been developed for LMD of root tissues in order to identify the most appropriate fixation and embedding conditions that preserve cellular morphology, while still enabling extraction of high quality RNA for PCR amplification. In this way, laser microdissected cells can be used for RNA extraction and expression studies, thus avoiding the dilution effect of RNA samples extracted from whole roots. In this work, the protocol previously developed [43] for the isolation of cells from tomato roots was applied for the acquisition of rice root cells. The use of paraffin tissue preparations coupled to Methacarn fixation provided rice root tissues that satisfactorily retain the cellular morphology. Next, RNA samples of high quality were obtained from laser microdissected root cells.

**Figure 2:**
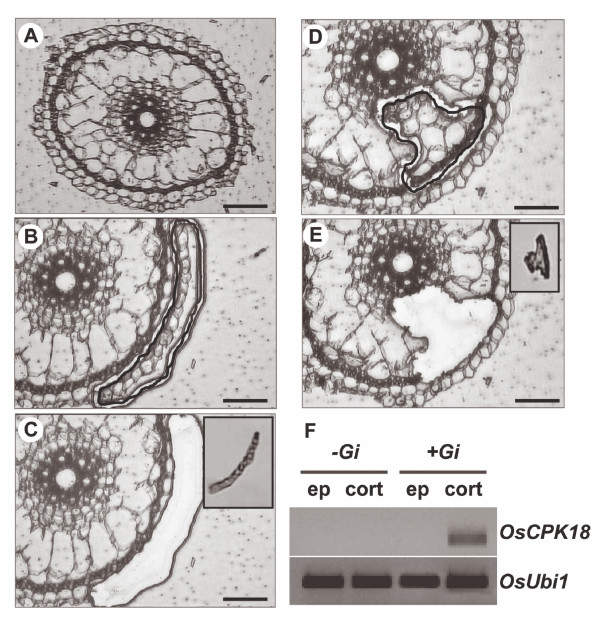
**Laser microdissection of epidermal and cortical cells from rice roots**. (A) Typical transverse section from the rice root. (B and C) Representative transverse sections with targeted epidermal cells before (B) and after (C) cutting with the laser microdissector. (D and E) Representative transverse sections with targeted cortical cells before (D) and after (E) cutting with the laser microdissector. Insets in C and E show captured microdissected cells. Two independent experiments were carried out for isolation of epidermal and cortical rice root cells by LMD. (F) RT-PCR analysis to detect *OsCPK18 *transcripts in laser microdissected cells from rice roots. Cells were harvested from *G. intraradices*-inoculated (+*Gi*) and mock-inoculated (-*Gi*) roots. Total RNA samples were obtained from pooled microdissected cells. Expression analysis was carried out using the one-step procedure for RT and PCR amplification. *OsCPK18 *transcripts were detected only in cortical cells. Bars, 100 μm (A), 50 μm (B to E).

Sections of the epidermis and the cortex were prepared from *G. intraradices*- and mock-inoculated rice roots at four days after inoculation (Figure 2). Cells, either epidermal or cortical cells, were collected pooled and used for RNA extraction. The cell type-specific pattern of expression of the *OsCPK18 *gene was examined in laser microdissected cells. As it is shown in Figure 2F, *OsCPK18 *transcripts were exclusively detected in cortical cells of *G. intraradices*-inoculated rice roots. *OsCPK18 *transcripts were not detectable in epidermal cells of the fungal-inoculated roots. The absence of PCR amplification products in epidermal cells of the fungal-inoculated roots was confirmed by nested PCR (results not shown). Transcripts for the *ubiquitin1 *gene (Figure 2F) or the *cyclophilin *gene (results not shown) were also detected in all the RNA samples obtained from laser microdissected cells. The use of gene-specific primers that span introns excluded the possibility of genomic DNA in total RNA samples used for RT-PCR analyses. The absence of an amplified product in RT-negative reactions also excluded any DNA contamination in RNA samples obtained from laser microdissected cells (results not shown). Finally, *OsCPK4 *transcripts were detected in RNA samples obtained from the two cell types captured from fungal-inoculated and control roots, this observation further supporting the integrity of the RNA samples used in this study (Additional file 2: Figure S2).

When comparing the results obtained on *OsCPK18 *expression in laser microdissected cells (Figure 2F) and whole roots (Figure 1), an apparent contradiction is observed. Thus, *OsCPK18 *transcripts were not detected in isolated cells from mock-inoculated roots (Figure 2F) whereas RT-qPCR analysis revealed *OsCPK18 *expression in whole roots (Figure 1A, upper panel). This finding could be explained taking into account the plant material and experimental approach used in these studies. In this work, only two cell types of the root were harvested for LMD-related analyses (epidermal and cortical cells). Thus, the detection of *OsCPK18 *expression in whole mock-inoculated roots could be due to the presence of cell types constitutively expressing *OsCPK18 *that were not analyzed with LMD (i.e. cells from the central cylinder). Additionally, transversal sections were routinely made at aprox. 2 cm from the root tip. Thus the observed expression of the *OsCPK18 *gene in regions of the rice root other than that used for laser microdissection (i.e. meristems) might well account for the observed *OsCPK18 *expression in mock-inoculated whole roots. This observation also illustrates the fact that results obtained in gene expression by using entire roots might often be misinterpreted and spatial differences in gene expression might not be perceived by using whole roots. Clearly, a more detailed analysis of *OsCPK18 *expression during growth and development of the rice root is needed.

### Subcellular localization of OsCPK18

**Figure 3:**
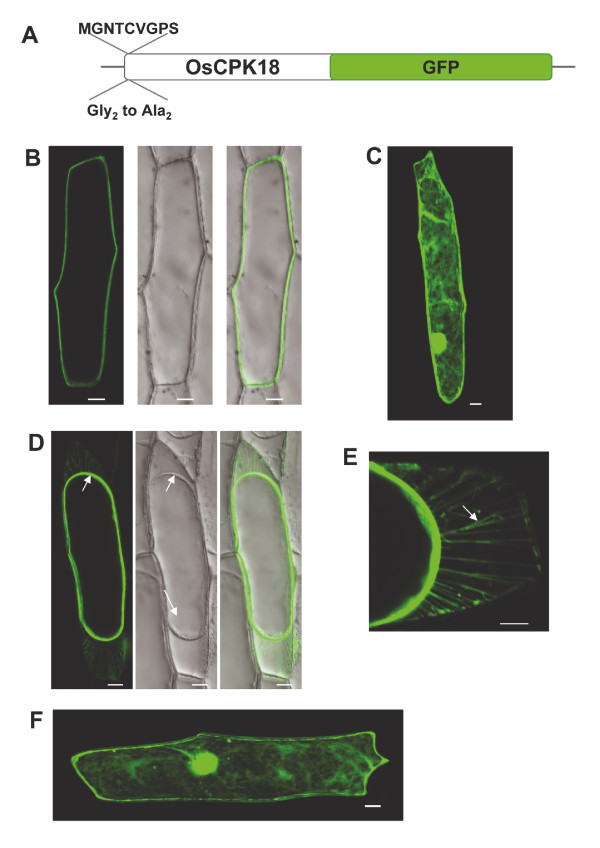
**Plasma membrane localization of OsCPK18**. Wild-type OsCPK18 and a N-terminal myristoylation mutant were transiently expressed as GFP fusion proteins in onion epidermal cells. Confocal images were taken 24 h post-bombardment. (A) Diagrams of the constructs used for particle bombardment of onion epidermal cells, wild OsCPK18-GFP and the mutant OsCPK18-GFP fusion protein in which the Gly_2 _was mutated to Ala (OsCPK18_G2A_). (B) Localization of wild OsCPK18-GFP fusion protein. Merged pictures of the green fluorescence channel with the corresponding light micrographs are shown in on the right. (C) Localization of GFP. (D) Onion cells after plasmolysis with mannitol (15 min of treatment). Light micrographs show the shrinkage of the protoplast (white arrow). (E) Treatment with mannitol renders the Hechtian strands (arrows) attaching the plasma membrane to the cell wall. (F) Onion epidermal cells expressing a mutated version of OsCPK18 with an altered myristoylation site (OsCPK18_G2A_-GFP). While the wild-type protein is localized to the plasma membrane, the G_2_A mutant protein lost its specific plasma membrane localization. Projection (B, C, D, F) and individual (E) sections are shown. Scale bars = 20 μm (B, C, D, F), 10 μm (E).

Onion epidermal cells are widely used as a convenient system in which to evaluate the subcellular location of GFP-tagged proteins. Accordingly, the subcellular localization of OsCPK18 was investigated in onion epidermal cells transiently expressing gene fusions to the green fluorescent protein (GFP) (Figure 3A). Confocal microscopy of transformed onion cells revealed that OsCPK18-GFP localizes to the cell periphery, likely the plasma membrane (Figure 3B). As expected, onion cells expressing the GFP gene showed fluorescence distributed throughout the cell (Figure 3C).

Onion epidermal cells are also particularly useful for analysis of plasma membrane proteins because the environmental conditions can be manipulated to cause plasmolysis and partial separation of the plasma membrane from the cell wall. The onion epidermal cells were plasmolyzed after being transformed with *OsCPK18-GFP*. In plasmolyzed onion cells, the OsCPK18-GFP displayed a pattern consistent with its location in the plasma membrane of the shrunken protoplasm (Figure 3D). Under these conditions, protoplast pull away from the cell wall, leaving large numbers of thin plasma membrane bridges, known as Hechtian strands, firmly anchored to the cell wall (Figure 3E).

Analysis of the amino acid sequence of OsCPK18 shows that the OsCPK18 polypeptide possess a N-terminal myristoylation site at the Gly residue at position 2 (Gly_2_) suggestive of N-myristoylation. The need for this lipid modification to promote and stabilize membrane association of certain CPKs has been experimentally demonstrated [37]. To address the role of the myristoylation site of OsCPK18 in plasma membrane association, a mutation at the N-terminal myristoylation site (MGNTCVGPS) of the OsCPK18 polypeptide was made. The Gly2 was converted to Ala (G2A, referred to as OsCPK18_G2A_) and fused to GFP (Figure 3A). Transient expression in epidermal onion cells showed that the Gly_2 _mutation abolished the plasma membrane localization of OsCPK18 (Figure 3F). Instead, a distribution throughout the cell was observed for the mutated version of OsCPK18 similar to that of the GFP alone. These findings suggest that the N-terminal myristoylation site is required for subcellular localization of OsCPK18 at the plasma membrane.

### Phylogenetic analysis of *cpk *genes

In this work, the evolutionary relationships among CPKs from rice and known CPKs from other plant species establishing association with AM fungi was determined. For this analysis, the full-length CPK protein sequences from cereal species, namely wheat and maize, as well as CPKs so far characterized in the model symbiotic species of *Medicago *were used. As previously mentioned, the rice genome contains 31 *CPK *genes which classify into four major phylogenetic groups (I-IV) [39,40]. Known CCaMK protein sequences from rice, wheat and *Medicago *were also considered. In this respect, the rice genome contains a single *CCaMK *gene [39]. As Arabidopsis is not a host for AM fungi, this species was not included in the phylogenetic analysis.

**Figure 4:**
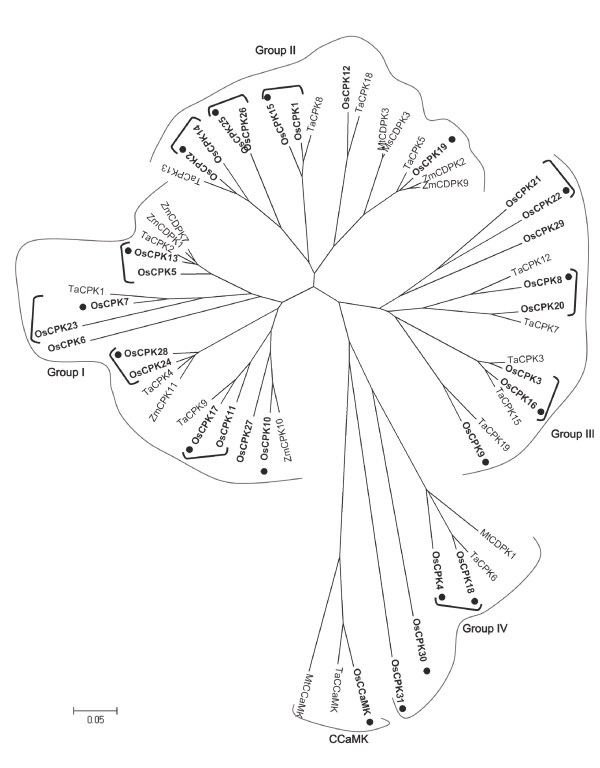
**Phylogenetic relationships among rice, wheat, maize and *Medicago *CPKs**. An unrooted phylogenetic tree was created using the MEGA4 program based on the full length sequences of CPK proteins from rice (Os), wheat (Ta), maize (Zm) and Medicago (*M. truncatula*, Mt; *M. sativa*, Ms). The four groups are indicated (I-IV). Rice CPKs are highlighted in bold. Members of each phylogenetic group of rice CPKs which have undergone expansion by segmental genome duplication (pairs of closely related CPKs) are indicated by brackets. Dots denote rice CPKs and CCaMK whose expression were analyzed in this work.

Phylogenetic trees of CPK and CCaMK proteins were constructed based on the neighbor-joining method (Figure 4) or the maximum parsimony method (Additional file 3: Figure S3). The aligment of the various proteins used for construction of the phylogenetic tree is presented in Additional file 4). Similar to what was previously reported [40], the rice CPKs clustered into four distinct phylogenetic groups (Figure 4). Four distinct CPKs, OsCPK18, OsCPK4, OsCPK30 and OsCPK31, cluster into an independent clade of CPKs, the Group IV, which appears to have diverged significantly from the other rice CPK sequences. Noticeably, results here presented show that *OsCPK18 *and *OsCPK4 *are both up-regulated by the AM fungus *G. intraradices*, these particular CPKs belonging to Group IV of rice CPKs. As for the other members of the Group IV of rice CPKs, no expression could be detected in the rice roots for *Oscpk31*, whereas *Oscpk30 *exhibited a low expression but no responsiveness to AM inoculation.

Some interesting observations came from the phylogenetic analysis of CPK and CCaMK proteins. Firstly, OsCPK18 and OsCPK4 appear to be closely related to the AM-associated MtCDPK1 (Figure 4). Secondly, Group IV of rice CPKs and CCaMKs are closely related each other. Indeed, Group IV of rice CPKs appears to be more related to CCaMKs than to the other rice CPKs. Here, it is worthwhile to mention that the essential function of *MtCCaMK *and *OsCCaMK *during the mycorrhizal symbiotic association is well documented [18,23]. Finally, the OsCPK18 is clearly related to TaCPK6, one of the 20 CPKs described in wheat [45].

### Sequence analysis of the *OsCPK18 *and *OsCPK4 *promoters

Knowing that the *OsCPK18 *and *OsCPK4 *genes are transcriptionally activated in response to inoculation with the AM fungus *G. intraradices*, it was of interest to investigate whether symbiosis-related *cis*-elements are present in the promoter region of these genes. The *OsCPK18 *and *OsCPK4 *promoter analysis was carried out using the PLACE algorithm [46] and extended to genes that are known to be required for both AM and rhizobial root nodule symbioses, such as the *MtCPK1 *and *MtCCaMK *genes from *M. truncatula *and the *OsCCaMK *from rice.

**Figure 5:**
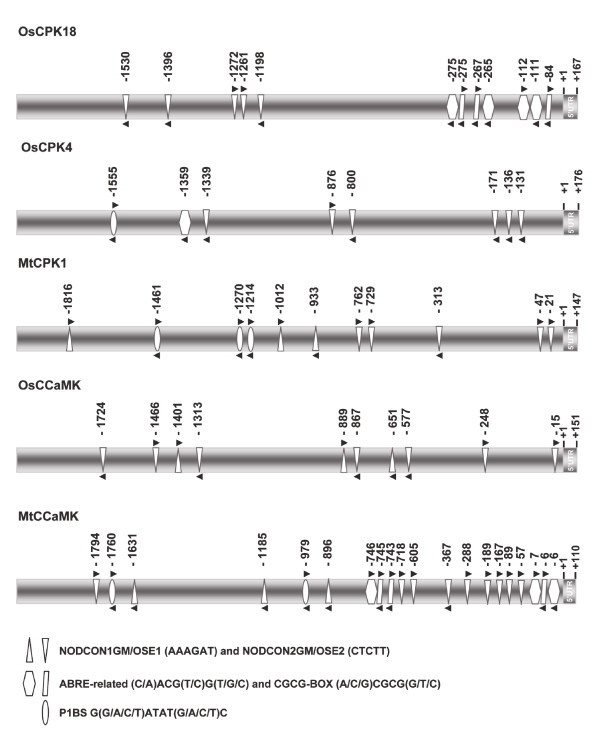
**Structural features of the promoters from the *OsCPK18*, *OsCPK4*, *MtCPK1*, *OsCCaMK *and *MtCCaMK *genes**. The location of the indicated *cis*-acting elements is indicated in each promoter.

Analysis of the 2 kb promoter region of the *OsCPK18 *and *OsCPK4 *genes revealed the presence of the CTCTT element (NODCON2GM) which is found up to five and six times in the *OsCPK18 *and *OsCPK4 *promoter, respectively (Figure 5 and Additional file 5: Tables S1 and S2). The NODCON2GM as well as the NODCON1GM element (AAAGAT) are characteristic motifs of promoters from genes that are regulated during root nodule and AM symbiosis. These motifs are also part of the "organ-specific element" (OSE) sequence [47]. The *MtCPK1, OsCCaMK and MtCCaMK *promoters contain several copies of the NODCON1GM and NODCON2GM consensus sequences.

Interestingly, multiple copies of the ABRE-related consensus motif [(C/A)ACG(T/C)G(T/G/C), ABRERATCAL] were present in the proximal region of the *OsCPK18 *promoter (Figure 5 and Additional file 5: Tables S1 and S2). The ABRE-related motif is a *cis*-element identified in the upstream region of 162 Ca^2+^-responsive up-regulated genes [48]. Furthermore, up to three copies of the CGCG-BOX element (GCCGCGGC) are found in the *Oscpk18 *promoter, this element being involved in Ca^++^/calmodulin-regulated gene expression [49] (Figure 5 and Additional file 5: Tables S1 and S2). The *OsCPK4 *promoter region contains one copy of the ABRE-related motif element. The G(G/A/C/T)ATAT(G/A/C/T)C (P1BS element) was recognized in the *OsCPK4, MtCPK1 *and *MtCCaMK *promoters (Figure 5 and Additional file 5: Tables S1 and S2). This element is found in the upstream region of phosphate starvation responsive genes from several plant species [50].

Finally, the *OsCPK18 *and *OsCPK4 *promoters harbor multiple stress-related *cis*-acting elements, including elements that are known to confer responsiveness to pathogen-regulated genes. Some of them were represented many times in these promoters, such as the TGAC-containing W box of WRKY transcription factors (Additional file 5: Tables S1 and S2). In line with this, we recently reported the activation of defense-and stress-related genes during colonization of rice roots by *G. intraradices *[4]. Whether the expression of the *OsCPK18 *and *OsCPK4 *genes is regulated during pathogen infection in roots remains to be determined.

Overall, this study revealed the presence of symbiotic-related motifs, as well as putative elements related to Ca^2+ ^regulation of gene expression, in the promoter region of the *OsCPK18 *and *OsCPK4 *genes. This observation is consistent with the observed induction for the two *CPK *genes in AM-inoculated rice roots.

## Discussion

In this work, the expression of *CPK *genes was monitored during the early stages of the AM symbiosis in rice. The *OsCPK18 *and *OsCPK4 *consistently showed up-regulation in response to AM inoculation. Evidence is also presented on the transcriptional activation of *OsCPK18 *and *OsCPK4 *expression by diffusible molecules produced by *G. intraradices*. When comparing the expression profiles of the rice *CPK *and C*CaMK *genes, it appears that activation of the two *CPK *genes (*OsCPK18 *and *OsCPK4*) occurs earlier than that of *OsCCaMK *pointing to a role for these particular rice *CPK *genes at the early stages of the symbiotic process. The observation that the *OsCPK18, OsCPK4, OsCCaMK, MtCPK1 *and *MtCCaMK *genes share symbiotic-related *cis*-elements in their promoters is also indicative of the transcriptional regulation of these genes as part of the signaling mechanisms involved in the AM symbiosis in rice. An expanded view of *OsCPK18 *gene expression came from expression studies in laser microdissected cells isolated from rice roots. At 4 days post-inoculation with *G. intraradices*, *OsCPK18 *was detected in cortical cells and not in epidermal cells.

Clearly, the specificity of a CPK functioning in a given signaling pathway may be achieved not only by a differential pattern of expression but also by targeting of the CPK protein to a particular subcellular compartment. Along with this, CPK proteins appear to be widely distributed among subcellular compartments including cytosol, peroxisome, plasma membrane, oil bodies and nucleus, as well as in association with actin filaments, mitochondria and the endoplasmic reticulum [33]. Our results in transformed onion cells clearly demonstrated that OsCPK18 localizes to the plasma membrane. Moreover, the association of OsCPK18 to the plasma membrane is possibly linked to N- terminal myristoylation of this protein.

Knowing that CPKs act as Ca^2+ ^sensors in plant signaling, and that Ca^2+ ^plays an important role in the AM symbiosis, a function of OsCPK18 as a Ca^2+ ^sensor during the AM-induced host responses to AM fungi can be envisaged. Thus, perception of the fungal-produced symbiotic signal(s) would activate downstream signaling events required for the establishment of the symbiotic association, including the cytoplasmic and nuclear Ca^2+ ^spiking responses [9-11]. Alterations in the Ca^2+ ^level would be itself a major factor in mediating up-regulation of *OsCPK18 *gene expression in the nucleus, as judged by the presence of the Ca^2+^-responsive *cis*-elements in the *OsCPK18 *promoter region [48]. In line with this, previous studies in Arabidopsis revealed the presence of ABRE-related sequences in Ca^2+^-responsive genes, and exclusively in up-regulated Ca^2+^-responsive genes [48]. Tetramers of the ABRE-cis element are sufficient to confer this transcriptional activation in response to Ca^2+ ^transients. The presence of multiple Ca^2+^-responsive *cis*-regulatory elements in the promoter region of the *OsCPK18 *gene (e.g. ABRE-related and CGCG-box elements) favors the possibility of a Ca^2+^-mediated up-regulation of *OsCPK18 *gene expression. The identity of the transcription factors that respond to rapid transient Ca^2+ ^signals and that subsequently activate gene expression through ABRE-related *cis-*elements remains to be determined.

In addition to its transcriptional activation, a direct regulation of the OsCPK18 enzyme activity by Ca^2+ ^can be expected. Thus, it is well known that the activity of CPKs is regulated by the binding of calcium to its intrinsic calmodulin-like domain. At basal Ca^2+ ^concentrations, the functional autoregulatory domain acts as a pseudosubstrate that inhibits the kinase activity of CPKs (autoinhibited structure). In response to transient increases in the level of cellular Ca^2+^, CPKs undergo conformational changes that activate their kinase activity (calcium-bound structure) [51]. It is then reasonable to assume that the plasma membrane-localized OsCPK18 protein sense the AM-induced increase in cytoplasmic Ca^2+ ^levels and transduce this signal into phosphorylation processes. The *OsCPK18*-mediated signaling processes might then be crucial for root colonization and accommodation of the fungal symbiont in the root cortex. The identification of downstream targets of the OsCPK18 kinase activity requires, however, further investigation.

On the other hand, our phylogenetic analysis of CPKs and CCaMK of plant species that are able to establish mycorrhizal associations revealed that Group IV of CPKs and CCaMK are closely related each other pointing to an evolutionary relationship between the two families of protein kinases. In other studies carried out in the green alga it was proposed that CCaMK originated through gene duplication from CPK during green alga evolution [52] Altogether, these findings are in clear support a functional specialization of members of the Group IV of CPKs and their relatedness with CCaMK functioning. Adaptation steps probably occurred in different plant species that determined their functional specialization and symbiosis-specific regulation.

The current work also provides a foundation for further functional investigation of the complex CPK family in relationship to the mycorrhization ability in another cereal species, such as wheat. Thus, the phylogenetic analysis of CPKs revealed that OsCPK18 and OsCPK4 are closely related to the wheat TaCPK6 protein as well as to the *Medicago *MtCDPK1 protein. For MtCDPK1 a role during the establishment of the AM symbiosis is well documented [53]. It is then tempting to speculate that the *TaCPK6 *gene might exhibit an AM-regulated expression pattern in wheat plants.

An intriguing aspect is the presence of three Arabidopsis proteins in Group IV of CPKs [39], even though Arabidopsis is not a host for AM fungi. To this point, it has been proposed that genes required for other aspects of plant development might have been recruited to function in symbiotic pathways. In line with this, inactivation of the *MtCDPK1 *gene is associated to a significant reduction of rhizobial and mycorrhizal symbiosis and also results in stunted roots and short root hairs in *M. truncatula *[53]. In other studies, impairment of root hair development results in defective symbiotic interactions in *L. japonicus *[54]. Then, the Arabidopsis CPKs within Group IV of CPKs might play a role in normal processes during root growth and development. The finding of SYM genes in species that do not associate with AM fungi (e.g. Arabidopsis and *Physcomitrella*), also supports that specific genes functioning in normal developmental processes in roots might also regulate mycorrhizal infection. If so, this fact, would explain the observed expression of OsCPK18 in experiments carried out on whole roots by RT-qPCR.

## Conclusions

This study provides a new view of the molecular mechanisms involved in the AM symbiosis in rice while defining an *OsCPK18*-mediated signaling pathway functioning during this process. The rapid activation of *OsCPK18 *expression in response to AM inoculation, its expression being also induced by fungal-secreted signals, together with the observed plasma membrane localization of OsCPK18, suggest that OsCPK18 might play a role in perception and/or recognition of the AM fungus in rice. Compared to legume species, less effort has been invested in the characterization of the AM symbiotic interaction in this important crop species. OsCPK18 might be considered as a marker for the presymbiotic phase of the symbiotic process that might play a preferential role in the root cortex. The identification of additional components of the AM-induced signaling processes in which OsCPK18 participates can be now approached. A major challenge for the future research is to determine whether interconnections and synergistic functions exist between CPKs and SYM components, this interplay determining recognition and compatibility between the two symbiotic partners.

## Methods

### Plant material and growth conditions

Rice (*Oryza sativa *cv Nipponbare) was used as the experimental material. Seeds were surface sterilized with 70% ethanol for 1 min, sodium hypochlorite (30% v/v) for 30 min, and extensively washed with sterile water (four times, 10 min each). Seeds were germinated in agar (0,4%) prepared with minimal medium. Seedlings were grown at 27°C ± 2°C under 18 h/6 h light/dark on vertical plates.

*G. intraradices *(DAOM197198) spores were prepared from monoxenic cultures of carrot roots that were grown for three months as previously described [4]. Roots and *G. intraradices *cultures were axenically solubilized in 5 volumes of sterile 10 mM sodium-citrate, pH 6.0 for 15 min, at 37°C and filtrated four times through a 250 μM sieve. Rice roots were inoculated with the *G. intraradices *spore suspension using either the single sandwich [29] or the double sandwich [42] system.

For the single sandwich method, the rice seedlings were directly inoculated with the arbuscular mycorrhizal spore suspension, or mock-inoculated (sterile water), and placed between two sterile nitrocellulose membranes (Millipore, pore diameter 0.45 μm). For the double sandwich method, the rice seedlings were first placed between two Millipore membranes. The membrane-covered seedlings were then inoculated with the fungal spore suspension and covered with a second layer of membranes. In this way, the physical contact between the fungus and the root is avoided. The assembled sandwich containing the inoculated seedlings was placed in Petri dishes containing 0.4% agar in minimal medium. Since Millipore membranes are permeable to diffusible molecules, the root cells can perceive fungal signals in the double sandwich method even thought physical contact between the two symbionts does not occur. Control seedlings were inoculated with sterile water.

### Tissue preparation and laser microdissection

The method previously described [43] was adapted for the isolation of cells from *G. intraradices*-inoculated and mock-inoculated rice roots. Root pieces of 4 - 8 mm in length were dissected with a razor blade and immediately transferred into freshly prepared Methacarn solution (absolute methanol/chloroform/glacial acetic acid 6:3:1). Roots were maintained in the fixative solution overnight at 4°C, and subsequently dehydrated in a graded series of ethanol at 4°C: 50, 70 and 90% in sterile water and 100% ethanol, followed by isopropanol (twice), with each step on ice for 1 h. The isopropanol was replaced gradually with paraffin (Paraplast Plus; Sigma Aldrich, St. Louis). Transverse root sections of 10-15 μm were made using a Reichert Jung 2050 SuperCut Motorized Microtome (Leica, Arnsberg, Germany). Ribbons were arranged on RNase-free, UV-treated, PEN-membrane 2.0 μm slides. Slides were kept in a slide warmer at 40°C until dry and stored at 4°C and used within two days.

The Leica LDM6000 Laser Microdissection system (Leica, Bannockburn, IL, USA) was used for laser microdissection (LMD). Just before use, the paraffin sections were deparaffinised in a neoclear (Merck, Darmstadt, Germany) treatment for 10 min followed by 100% ethanol for 2 min, and then air dried. The deparaffinised slides were placed face down on the microscope. The tissues were visualized on a computer monitor through a video camera. Epidermal and cortical cells were marked and then cut using a UV laser (337-nm wavelength). Target cells were collected without any extra forces into the cap of a microcentrifuge (RNase-free PCR tube caps). For each cell type, we isolated at least 1500 cell sections per biological replicate, and two independent biological replicates were made. After collection, 50 μl of RNA extraction buffer from the PicoPure kit (Arcturus, Sunnyvale, CA, U.S.A.) were added. Samples were incubated at 42°C for 30 min, centrifuged at 800 g for 2 min, and stored at -80°C until RNA isolation.

### RNA isolation

Total RNA was extracted from whole roots at different times after inoculation with *G. intraradices *spores, as well as from mock-inoculated roots, using the TRIZOL^®^Reagent (Invitrogen, Carlsbad, CA, USA). For each time point, roots from at least 12 individual plants were collected. Three independent experiments were carried out. The first cDNA was synthesized from DNase-treated total RNA (1 μg) with M-MLV (Moloney-Murine Leukemia Virus) Reverse Transcriptase (Invitrogen, Carlsbad, CA, USA). Aliquots of the resulting RT reaction product were used as template for PCR analysis.

RNA isolations from laser microdissected cells were performed using a modified PicoPure kit protocol (Arcturus, Sunnyvale, CA, USA). Essentially, the DNase-treatment was not performed on the kit column but the RNA was eluted in 25 μl of DEPC-treated H_2_O and then treated with RNAse-free DNase (Roche, Mannhein, Germany). After precipitation with 0.1 vol. of 3M Na-acetate and 2.5 vol. cold ethanol (100%), the RNA was resuspended in 20 μl of sterile water and quantified using the NanoDrop 1000 spectrophotometer.

### Gene expression analysis

Quantitative real time PCR (RT-qPCR) analyses were carried out in optical 96-well plates in a LightCycler^® ^480 Real-Time PCR System (Roche) according to the following program: 10 min at 95°C, followed by 45 cycles of 95°C for 10 s, 60°C for 30 s, and an additional cycle of dissociation curves to ensure an unique amplification. The reaction mixture contained 10 μl 2× SYBR Green Master mix reagent (Roche, Mannhein, Germany), 2 μl cDNA sample, and 300 μM of each gene-specific primers, in a final volume of 20 μl. Primers used for RT-qPCR are indicated in Additional file 6: Table S3. Details on RT-qPCR analysis following the MIQE guidelines [55] are included in Additional file 7. Routinely, three replicate reactions were used for each sample. Data were normalized with *OsAct1 *as internal control. The average CT values from triplicate PCRs were normalized to the average CT values for the *OsAct1 *gene from the same RNA preparations. Three independent biological replicates were analyzed. For each biological material, three technical replicates were made for RT-qPCR analysis.

In this work, semi-quantitative reverse-transcription-polymerase chain reaction (RT-PCR) was carried out to investigate the expression pattern of the rice *CPK *genes, as well as the rice *SYM *genes, namely the *OsSYMRK, OsCCaMK, OsPOLLUX *and *OsCASTOR *genes. (Additional file 8: Table S4 and Additional file 9: Supplementary Methods).

For RT-PCR analysis of RNA samples obtained from laser microdissected cells, a one-step RT-PCR was conducted according to the manufacturer's instructions (Qiagen GmbH, Hilden, Germany). A 20 μl reaction was prepared, containing the following reagents for each reaction: 4 μl of (5X) Qiagen one-step RT-PCR buffer, 0,5 μl of Qiagen one-step RT-PCR enzyme mix, 0,5 mM of each dNTP, 0,25 μM of each primer, 21 ng of total RNA and RNase free water to 20 μl.

### Phylogenetic analysis

The full length protein sequences were obtained from the NCBI database and aligned using ClustalW [56]. The alignment of the various plant CPKs was created with GeneDoc 2.7.0 Software [57] and is shown in Additional File 4.

In addition to the 31 CPKs from rice, known full-length CPK sequences from *M. truncatula*, *M. sativa *and *Zea mays *were also used. They were (accession numbers are from Gene Bank at the National Center for Biotechnology Information (NCBI) database http://www.ncbi.nlm.nih.gov/: MtCDPK1 [AAX15706], MtCDPK3 [ABE72958], and MsCDPK3, X96723]; ZmCDPK1 [BAA12338], ZmCDPK2 [AAA69507], ZmCDPK7 [BAA13232], ZmCDPK9 [BAA12715], ZmCDPK10 [CAA07481] and ZmCDPK11 [AAP57564]. As for wheat, up to 20 CPKs have been described from which 14 available sequences were included in this study [45]. They were: TaCPK1 [ABY59004], TaCPK2 [ABY59005], TaCPK3 [ABY59006], TaCPK4 [ABY59007], TaCPK5 [ABY59008], TaCPK6 [ABY59009], TaCPK7 [ABY59017], TaCPK8 [ABY59010], TaCPK9 [ABY59011], TaCPK12 [ABY59012], TaCPK13 [ABY59018], TaCPK15 [ABY59013], TaCPK18 [ABY59014], TaCPK19 [ABY59015]. Furthermore, CCaMK sequences from rice, wheat and *Medicago *were included in this analysis, the rice OsCCaMK [Q6AVM3], wheat TaCCaMK [ADK22086] and *M. truncatula *MtCCaMK [Q6RET7] sequences. Phylogenetic trees were created according to the neighbor-joining method (Figure 4) or the maximum-parsimony algorithm (Additional File 3: Figure S3) using MEGA4 program [58,59].

### Promoter analysis

Sequences 2000 bp upstream of the selected *cpk's *genes were retrieved from NCBI database. Known plant motifs were obtained from the PLACE database http://www.dna.affrc.go.jp/PLACE/.

### Biolistic cell transformation and imaging by confocal microscopy

To investigate the subcellular localization of OsCPK18, the green fluorescent protein (GFP) gene was translationally fused to the C-terminal end of the *OsCPK18 *sequence. For this, a *GFP *gene lacking the N-terminal signal peptide and C-terminal HDEL sequences was initially obtained by PCR from the *m-GFP5-ER *sequence [60]. Primers used were 5'-CGGGGATCCATGAGTAAAGGAGAAGAACTTTTCAC-3' (forward) and 5'-CCGAGCTCTTATTATTTGTATAGTTCATCCATGC-3' (reverse). During the PCR reaction, *BamHI *and *SacI *sites were introduced into the PCR amplified DNA fragment (underlined nucleotides in PCR primer sequences). Next, the *GFP *variant was cloned between the *BamHI *and *SacI *sites of a pUC19-derived plasmid harboring the *35SCaMV *promoter and nopaline synthetase terminator (*Tnos*) to create the *pP35S:mGFP:nos *construct.

The *OsCPK18 *DNA fragment to be translationally fused to the *GFP *gene was generated by PCR amplification. A *BglII *site was introduced at the 3'end of the PCR-amplified *Oscpk18 *DNA fragment. Equally, a *Spe*I site was introduced at the 5' end of the *Oscpk18 *sequence. The full-length *OsCPK18 *cDNA sequence was PCR-amplified from clone J023148F12 obtained from the KOME (Knowledge-based Oryza Molecular biological Encyclopedia) database using the following primers: 5'-ATACTAGTATGGGACTCTGCTCCTCCTCC-3' (forward) and 5'- ATAGATCT**ACCTGGTGG**TGGCGATCTGTGAACACTCCT-3' (reverse) (underlined sequences indicate the *SpeI *and *BglII *restriction sites; bold fonts indicate the residual tail of one glycine and three prolines to ensure the fusion). To obtain the chimeric *OsCPK18-GFP *gene, the PCR-amplified *OsCPK18 *DNA fragment was digested with *SpeI *and *BglII *and cloned into the XbaI/BamHI-digested *pP35S:GFP:nos *plasmid resulting in plasmid *pP35S:OsCPK18-GFP:nos*.

A chimeric gene in which a mutated version of *OsCPK18 *gene was fused to the *GFP *gene was also prepared. For preparation of the mutated version of the *OsCPK18 *gene in which the Gly residue at position 2 was converted to Ala (G2A), the *pP35S:OsCPK18-GFP:nos *plasmid was used as the template for PCR. For this, a forward primer with a single change (bold font) 5'-ATACTAGTATGG**C**ACTCTGCTCCTCCTCC-3', and the *OsCPK18 *reverse primer above indicated were used. The *OsCPK18*_*G2A *_DNA was then cloned into the *XbaI/BamHI*-digested *pP35S:mGFP:nos *plasmid. All the constructs were verified by nucleotide sequencing.

Onion epidermal cells were transformed by particle bombardment. The epidermis of onion bulb scales was prepared by peeling the inner epidermis from fresh onion bulbs. For each DNA construct, 4 μg of DNA was mixed with 20 μμl of 0.1 M spermidine, 50 μμl of 2.5 M CaCl_2 _and 15 μl of gold microcarrier (60 μg ml^-1 ^in 50% glicerol), vortexed vigorously for 30 sec and centrifuged at 800 g in a microfuge for 10 sec. The pellet was washed on ethanol. The DNA-gold particles were bombarded into cells at a pressure of 1100 psi and target distance of 6 cm, with a biolistic PSD-1000/He particle delivery system (Bio-Rad). The transfected cells were kept on MS medium for 16-24 h in the dark. The subcellular localization of the fusion proteins was determined by confocal laser scanning microscopy (CLSM) in a Olympus Fluoview FV1000 confocal laser scanning microscope (Tokyo, Japan). The excitation wavelength was 488 nm. The emission window was set at 500-600 nm for GFP. To confirm plasma membrane localization, cells were plasmolysed with 0.75 M mannitol.

## Authors' contributions

LCS carried out most of the experimental work and data analyses. JGA participated in laser microdissection and gene expression studies. BSS coordinated the design and execution of this study and wrote the manuscript. PB also participated in the design of this study and critically revised the manuscript for important intellectual content. All authors read and approved the final manuscript.

## Supplementary Material

Additional file 1 **Figure S1: RT-PCR analysis of rice *CPK *genes in mock-inoculated (-) and *G. intraradices*-inoculated (+) rice roots**. Inoculation with *G. intraradices *spores was carried out using the single-sandwich system. Roots were harvested at different times after inoculation and each RNA sample was prepared from a pool of roots from 12 plants. RT-PCR was performed using specific primers for the indicated *CPK *genes. The subset of genes selected for expression analysis included members of the four phylogenetic groups of *CPK *genes (I-IV) (for details see Figure 4). Expression of rice SYM marker genes, the *OsCCaMK, OsCASTOR*, *OsPOLLUX *and *OsSYMRK *genes was analyzed in the same RNA samples that were used for analysis of *CPK *gene expression. Transcripts for the *OsCPK2*, *OsCPK22*, *OsCPK25 *and *OsCPK31 *could not be detected (results not shown, similar results were reported previously [40] by microarray analysis). The constitutively expressed *actin1 *(*OsAct1*) gene was used as the internal control in these experiments. Three independent experiments were carried out with similar results. Click here for file

Additional file 2 **Figure S2: Expression of the *OsCPK4 *gene in laser microdissected cells from rice roots**. Cells were harvested from *G. intraradices*-inoculated (+*Gi*) and mock-inoculated (-*Gi*) roots. Total RNA samples were obtained from pooled microdissected cells. Expression analysis was carried out using the one-step procedure for RT and PCR amplification. Although different expression levels were observed in epidermal and cortical cells, RT-PCR analyses from RNA samples obtained from laser microdissected cells does not provide quantitative information on gene expression. Click here for file

Additional file 3 **Figure S3: Relationship between plant CPKs**. The phylogenetic tree was constructed using the maximum parsimony method using the full length amino acid sequences of CPKs from rice (Os), wheat (Ta), maize (Zm) and Medicago (*M. truncatula*, Mt; *M. sativa*, Ms) and CCaMKs. The four groups are indicated (I-IV). Click here for file

Additional file 4 **Alignment of the amino acid sequences of plant CPKs and CCaMKs**. Amino acid sequences from rice (Os), wheat (Ta), maize (Zm) and Medicago (*M. truncatula*, Mt; *M. sativa*, Ms) were aligned. Black and gray backgrounds indicate amino acids that are identical or similar, respectively. Dashes indicate no residue present at that position. The boundaries of the kinase (amino acids 51-317 in OsCPK18) and calmoldulin-like (amino acids 347-493 in OsCPK18) domains are shown (black and grey arrows, respectively). Click here for file

Additional file 5 **Table S1 and Table S2**. Table S1. Symbiosis-related *cis*-elements identified in the *OsCPK18*, *OsCPK4*, *MtCPK1*, *OsCCAMK *and *MtCCaMK *promoters. The 2 kb upstream region of each promoter was analyzed by using the PLACE motif database. Table S2. Number of symbiosis-related *cis*-elements present in the *OsCPK18, OsCPK4*, *MtCPK1*, *OsCCaMK *and *MtCCaMK *promoters. The presence (x) or absence (-) of the indicated motifs within the 2 kb upstream region of each promoter is shown. Multiple copies of a given *cis*-element were present in a particular promoter (2x, 3x, 4x, etc.). The PLACE motif database was used to perform this analysis. Motifs are listed by alphabetical order. Click here for file

Additional file 6 **Table S3: Primer sequences used in the real-time qPCR analysis**. Click here for file

Additional file 7 **Information of the RT-qPCR analysis based on the MIQE (Minimum Information for Publication of Quantitative Real-Time PCR Experiments) checklist **[55]. Click here for file

Additional file 8 **Table S4: Primer sequences used in the RT-PCR analysis**. Click here for file

Additional file 9 **Supplementary Methods**. Gene expression analysis by semi-quantitative RT-PCR. Click here for file
