# Supplementary figures and images for: A rice calcium-dependent protein kinase is expressed in cortical root cells during the presymbiotic phase of the arbuscular mycorrhizal symbiosis
Source: BMC Plant Biol. 2011 May 19;11:90. doi: 10.1186/1471-2229-11-90 (PMC3125349; doi:10.1186/1471-2229-11-90)

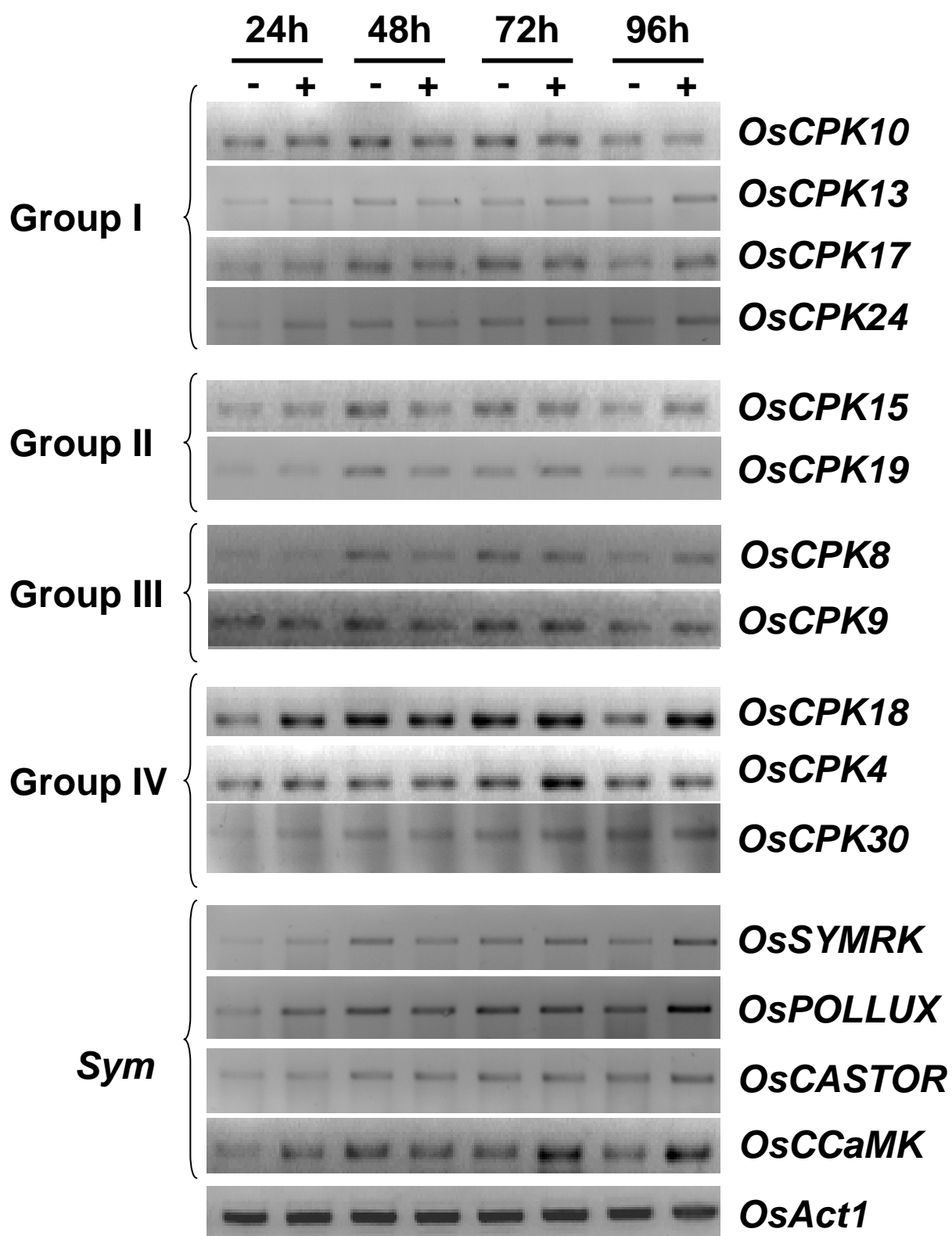

Figure S1

Supplement: Additional file 1 — Figure S1: RT-PCR analysis of rice CPK genes in mock-inoculated (-) and G. intraradices-inoculated (+) rice roots. Inoculation with G. intraradices spores was carried out using the single-sandwich system. Roots were harvested at different times after inoculation and each RNA sample was prepared from a pool of roots from 12 plants. RT-PCR was performed using specific primers for the indicated CPK genes. The subset of genes selected for expression analysis included members of the four phylogenetic groups of CPK genes (I-IV) (for details see Figure 4). Expression of rice SYM marker genes, the OsCCaMK, OsCASTOR, OsPOLLUX and OsSYMRK genes was analyzed in the same RNA samples that were used for analysis of CPK gene expression. Transcripts for the OsCPK2, OsCPK22, OsCPK25 and OsCPK31 could not be detected (results not shown, similar results were reported previously [40] by microarray analysis). The constitutively expressed actin1 (OsAct1) gene was used as the internal control in these experiments. Three independent experiments were carried out with similar results. [file 1471-2229-11-90-S1.PDF]

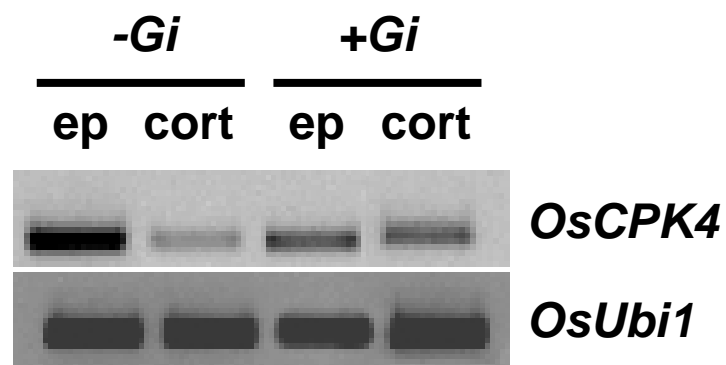

**Figure S2**

Supplement: Additional file 2 — Figure S2: Expression of the OsCPK4 gene in laser microdissected cells from rice roots. Cells were harvested from G. intraradices-inoculated (+Gi) and mock-inoculated (-Gi) roots. Total RNA samples were obtained from pooled microdissected cells. Expression analysis was carried out using the one-step procedure for RT and PCR amplification. Although different expression levels were observed in epidermal and cortical cells, RT-PCR analyses from RNA samples obtained from laser microdissected cells does not provide quantitative information on gene expression. [file 1471-2229-11-90-S2.PDF]

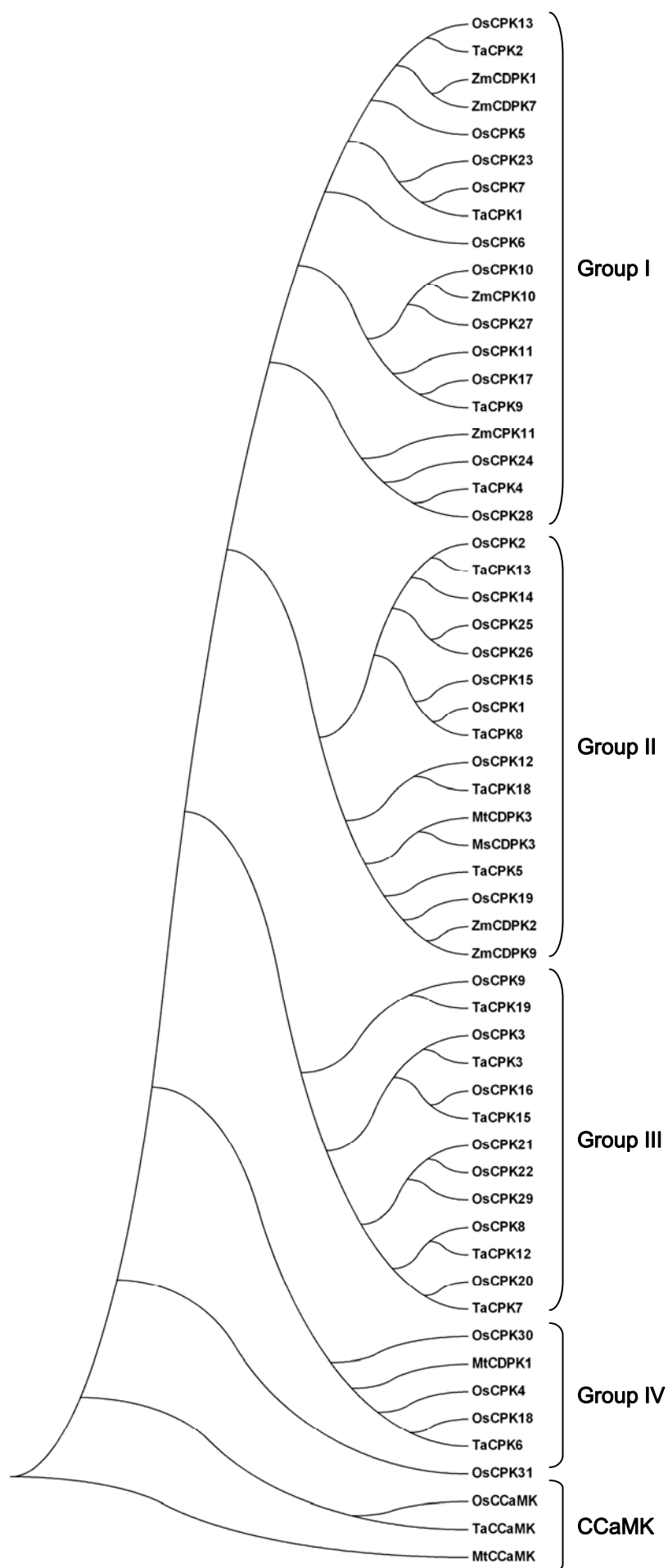

**Figure S3**

Supplement: Additional file 3 — Figure S3: Relationship between plant CPKs. The phylogenetic tree was constructed using the maximum parsimony method using the full length amino acid sequences of CPKs from rice (Os), wheat (Ta), maize (Zm) and Medicago (M. truncatula, Mt; M. sativa, Ms) and CCaMKs. The four groups are indicated (I-IV). [file 1471-2229-11-90-S3.PDF]
