# Supplementary material for: A rice calcium-dependent protein kinase is expressed in cortical root cells during the presymbiotic phase of the arbuscular mycorrhizal symbiosis
Source: BMC Plant Biol. 2011 May 19;11:90. doi: 10.1186/1471-2229-11-90 (PMC3125349; doi:10.1186/1471-2229-11-90)
Supplement: Additional file 4 — Alignment of the amino acid sequences of plant CPKs and CCaMKs. Amino acid sequences from rice (Os), wheat (Ta), maize (Zm) and Medicago (M. truncatula, Mt; M. sativa, Ms) were aligned. Black and gray backgrounds indicate amino acids that are identical or similar, respectively. Dashes indicate no residue present at that position. The boundaries of the kinase (amino acids 51-317 in OsCPK18) and calmoldulin-like (amino acids 347-493 in OsCPK18) domains are shown (black and grey arrows, respectively). [file 1471-2229-11-90-S4.PDF]

```
OsCPK18  MGLCS--------------------------SSSARRDAGTPGGGNGAGNKDNA---------------------------GRKG-----------------------------------------------------------   32
TaCPK6   MGLCT--------------------------SSSAASPAG-PAGNKEKGRKGSG---------------------------GCRG-----------------------------------------------------------   31
MtCDPK1  MGLCF--------------------------SSTKVVSGSNSNTTNNDNRKRNQSTTTDTTVTVTTATTAAQK----QTAQRRKGGSNETA------------------QKKNHHQHHRLKEKTG-------------------   77
OsCPK4   MGACF--------------------------SSHTATAAADGGSGKRQQRKGDHKG---------------------KLPDGGGG-----------------------------------------------------------   38
OsCPK30  MGLCHGKPSQIPEPEAEEAAAAAGVAVAGAASPGPAAAAAAAAAAKPGTPKPQKPFPFYLPSPLPASSYKSSPANSSVASTPARGGLKRPFP------------------PPSPAKHIRALLARRHGSVK---------------  111
OsCPK3   MGN---------------------------CCRSPAAAAREDVKSSHFPASAG-----------------------KKKPHQARNGGVGGG--------------------GGGGGGGGGGG----------------------   52
TaCPK3   MGN---------------------------CCRSPAAAAREDVKSSHFPASAAA-------------------AARKNKPHQPRSGAAGTG--------------------GGGG-----------------------------   49
OsCPK16  MGN---------------------------CCRSPAAAAREDVKTSHFPASTGG-------------------G--KKKPHQARNGGGGGG--------------------GGGGGG---------------------------   49
TaCPK15  MGN---------------------------CCRSPAAAAREDVSSSHFPASAG-----------------------KRKPHQARNGGGGGA--------------------GGGGGGGGAGA----------------------   52
OsCPK8   MGN---------------------------CCGTPATAEEGGKRRRRGKQ-------------------------KKANPFTVAYNRAPSS------------------AGAAAGR----------------------------   46
TaCPK12  MGN---------------------------CCGAPSTQG-GGRGKNDRRK----------------------------PKANPYTVAYNRG------------------AGAPPAR----------------------------   42
OsCPK20  MGN---------------------------CCVTP---EGSGRGRKKQQQEQKQKQKEPKQQ------QQQQKKGKKPNPFSIEYNRS-------------------------SAPSG--------------------------   57
TaCPK7   MGN---------------------------CCATPPAVDGGGGGGGKQQ-------KEPKQ-----------RKGKKPNPFSIEYNRS-------------------------APPGA--------------------------   48
OsCPK9   MGN--------------------------TCCVAPATT--DEVGAPPRDHHHAAKKSPAPSATTTTATRQRHGQEPKPKPKPRARAKPNPYDWAPP-----------RVLPARGGAAAS-------------------------   80
TaCPK19  MGNACF-----------------------SCCTASSQPELDAXGAPRPQHRKRPATPPRPSRSSEPSPTAARAS-PKPRPRTRAKAKPNPY-------------------AQRGGGGS--------------------------   75
OsCPK21  MGS---------------------------CYSAYASS----RKLRGRISKISLVIPDPVPD----------AEAASPRKDGVDGDGDD--------------------VRGGGGGCD--------------------------   57
OsCPK22  MGG---------------------------CSSAFAVSTRMIRFSRGRVPAAILPVTSNDEP----------CCSCSPENNNKNNDGGGGGCDGGEHQKGKSWRRWQYRRCGGGGGG---------------------------   80
OsCPK29  MGN---------------------------CCVSRPSGADKRRRCGSSTAPHTRGGRRVIGA----------ANMRCLSTVSSVSDAARAVMSNEP-----------ATVLGNSGSSG--------------------------   70
OsCPK1   MGNR--------------------------TSRHH------------------------RAAPEQPP----PQPKPKPQ---PQQQQQQWP------------------RPQQPTPPPA-------------------------   44
TaCPK8   MGNR--------------------------TSRHR------------------------RAAADQPA----TAPPPTAQ---PKPQPPPKP------------------QTAPAPAPTP-------------------------   44
OsCPK15  MGAR--------------------------ASRHRQSPDQSQSQSPSPHHKHHHHHQTTRAPKPKPK----PQPPPPQQ---PRSQPPPPP------------------RHQPQQAPQQ-------------------------   68
OsCPK2   MGNC--------------------------CP---------------------------GSGDAEP-----ASSDASTG---NGSSSFKAG-------------------ASPSSAPAQ-------------------------   39
TaCPK13  MGNC--------------------------CA---------------------------GSGDAEPA----AAADPSTR---RAGASVQAG------------------GASPSSAPAQ-------------------------   41
OsCPK14  MGNC--------------------------CPPG-------------------------SSSEPDPP----PASSGSSRPAGSAGAAASPA------------------TISPSAAPAP-------------------------   46
OsCPK25  MGQC--------------------------CTGGGKAVAGDEAEP--------------GTSKAAPPSRGTSSKNGSAKQQPCSPAAKAAA------------------TEAAAAASSS-------------------------   61
OsCPK26  MGQC--------------------------CTGGGKAVAGDEAEP--------------GTSKAAPPSRGTSSKNGSAKQQPCSPAAKAAA------------------TEAAAAASSS-------------------------   61
OsCPK12  MGNC--------------------------FTKTYEIPITSGTMRR-PASTAERSKARGGDEPG--TWRRPSFPRHGAPPHRP---PTGSS------------------SAAGALSRRA-------------------------   69
TaCPK18  MGNC--------------------------FTKAYEIPISSGSFKRPPPSFGEQPPPAGHGKPPRPPSRRSTFPKP-PPPPRPSGRPPLPS------------------FLSGSLSRKV-------------------------   74
OsCPK19  MGSC--------------------------CSRA-----TSPD------SGRGGANGYGYSHQTKPAQTTPSYNHP-QPPPPAEVRYTPSA------------------MNPPVVPPVV-------------------------   63
TaCPK5   MGQC--------------------------CSRA-----TSPD------SVQGGANGYGYSHQPKQAQTPPSYNNA-QPPPQAEVRYTPPA------------------MNPPVVPPVV-------------------------   63
ZmCDPK2  ---M--------------------------VMAI-----LTRQ------SRR---------------KHLRVYNPP-QQA--AEVRYTPSA------------------TNSSAVPPVA-------------------------   43
ZmCDPK9  MGQC--------------------------CSRA-----PAPD------SGRGGTNGYGYSNQAKPAQTPSSYNPP-QQP--AEVRYTPPA------------------TNPPVVPPVP-------------------------   61
MtCDPK3  MGCL--------------------------LSKD-----KDSEPEHNNGGYRYGE----HNHRNNHEQVNTSRTAP-TSSYQPQIPTKPSS------------------ISAPSPKPLI-------------------------   65
MsCDPK3  MGCL--------------------------LSKD-----KDSEQEHNNGGYRYAESSGMHNHRNNHEQVNTSRTAP-TSSYQPQIPTKPSS------------------ISAPSPKPLI-------------------------   69
OsCPK10  MGNT--------------------------CVGPS--ISKNGFFQSVS-TVLWKARQDGDDALPGANGAPDG-GGQGRLPAPPPPTSDAP-------------------LAVQNKPPEHVKIVS--------------------   75
ZmCPK10  MGNT--------------------------CVGPS--ITMNGFFQSVS-TALWKTPQEGD-ALPAAANGPGGPAGAGSQSALPKPASDVHH------------------VAVQSEAPEPVKIAAYHSEPAPAVRSEAPEPVKIA   96
OsCPK17  MGNT--------------------------CVGPSSAADRHGFFHSVSLAVLWR---------------PGG-RAEPSQPPGYPPRESSHS------------------SVTSSTAPERV-----------------------T   61
TaCPK9   MGNT--------------------------CAGPSATADRHGFFSTVSVAVLWR---------------PGAARAEPAVPP--PDSCPSMC------------------SSTSSAGPD--------------------------   57
OsCPK11  MGNN--------------------------CVGPS-AAGQNGFFANV---ALWR---------------PRPADAAPPALPPPSSAPSDQA------------------PEPVTIPPSEH-----------------------S   58
OsCPK27  MGNV--------------------------CIGPRRNFAKNGLLGILRPRHAAPSSPSQP--TTTSRSIPVVLPSAPSSKPPPPTQTAPPV------------------PVVISEPPPPQPQPEPQP--------------AAP   84
OsCPK24  ------------------------------------------------------------------------------MQPDP--SGSGGD------------------DNANAKAKLA-------------------------   21
TaCPK4   ------------------------------------------------------------------------------MQPDA--SGNAGG------------------GGANPRPKLP-------------------------   21
ZmCPK11  ------------------------------------------------------------------------------MQPDP--SGN-----------------------ANAKTKLP-------------------------   16
OsCPK28  ------------------------------------------------------------------------------MQPDPQPHGRGRE------------------KAAGAGPRLP-------------------------   23
OsCPK13  MGNA--------------------------CGGSLRSKYLS-FKQTAS-QRHDTD-DNNNAAAADSPKK-PSRPP---AAAKTDDHPVSAS------------------APAAAMRRG--------------------------   67
TaCPK2   MGNA--------------------------CGGSLRSKYLHSFKHPAS-QRHDPDRDYDHTAAADSPKKQPGSQPTATAAAKTDGHAAPA-------------------QPAAAMRRGG-------------------------   73
ZmCDPK1  ------------------------------------------------------------------------------------------------------------------MRRGG-------------------------    5
ZmCDPK7  MGNA--------------------------CGGALRSKHQHSFKHAASSQRHHASSEYSASAAADDSPRKPPPPP-----ATTDAHAPPP-------------------PPATAMRRGG-------------------------   69
OsCPK5   MGNT--------------------------CGVTLRSKYFASFRGASQRHDEAGYAPVATSAAAAAAADEPAGKK-----APRGSAAAADA------------------PHAASMKRG--------------------------   69
OsCPK7   MGNQ--------------------------C---QNGTLGSDYHN--RFPREHAVG----YVQGDSYLDLKKFDD------TWPEVNNFKP------------------TAASILRRG--------------------------   59
TaCPK1   MGN-------------------------------QNGTPGNDYCS--RFPREHPASRYADGIEDDSYSDLKKSEK------PWPDADSFKP------------------TAAGILRQG--------------------------   61
OsCPK23  MGNS--------------------------C---QNGTYGNNYQNSNRFQNDRFAS---------RYVDGNDTED------CYSGSS--RA------------------SLAGALRQG--------------------------   54
OsCPK6   MGNY--------------------------YSCGASSTSSPTSPSLVDYYYCYHR-----------YPSSCSSTS------TATSSGGRMP------------------IRSHQQRLS--------------------------   57
OsCPK31  ------------------------------MRGLVLQGRQQSLQGRLRSSSKFCVSRPVAPRPCKACRRAARSGSSSCSRAVAVQVVASLD------------------YIATASDEGVLKLP---------------------   75
OsCCaMK  ------------------------------------------------------------------------------------------------------------------------------------------------    -
TaCCaMK  ------------------------------------------------------------------------------------------------------------------------------------------------    -
MtCCaMK  ------------------------------------------------------------------------------------------------------------------------------------------------    -
```

```
OsCPK18  --------------------------------------------------IVACGKRTDFGYDKDFEARYALGKLLGHGQFGYTFAAVDR---RSSER---VAVKRIDKNK---------------------------MVLPVAVED   96
TaCPK6   --------------------------------------------------IIACGKRTDFGYDKDFEARYALGKLLGHGQFGYTFAAVDR---QSDER---VAVKRIDKNK---------------------------MVLPVAVED   95
MtCDPK1  ------------------------------------------------SKHVPCGKRTDFGYEKDFDKRFSLGKLLGHGQFGYTYVGVDK---SNGDR---VAVKRLEKAK---------------------------MVLPIAVED  143
OsCPK4   -------------------------------------------------EKEKEAARVEFGYERDFEGRYQVGRLLGHGQFGYTFAATDR---ASGDR---VAVKRIDKAK---------------------------MVRPVAVED  103
OsCPK30  -----------------------------------------PNEAPIPEGGETEVGLDKGFGSKHFFAKYELGDEVGRGHFGYTCSAKAK---KGDHKGHDVAVKVIPKAK---------------------------MTTAIAIED  188
OsCPK3   ---------------------------------------------GAGQKRLPVLGEEGCELIGGIDDKYALDRELGRGEFGVTYLCMDR---DTKEL---LACKSISKRK---------------------------LRTAVDVED  121
TaCPK3   ------------------------------------------------QKRLAVLGEEGCDFIGGIDDKYLLDRELGRGEFGVTYLCVDR---DTKEQ---LACKSISKRK---------------------------LRTPVDVED  115
OsCPK16  ----------------------------------------------WEKKRLSVLGEEGSEVNGGIEEKYALDRELGRGEFGVTYLCMDR---CSREL---LACKSISKRK---------------------------LRTPVDVED  117
TaCPK15  ---------------------------------------------GGEKKRLSVLGEEGPDVSGGIDDKYALDRELGRGEFGVTYLCMDR---GSKEL---LACKSISKRK---------------------------LRTPVDVED  121
OsCPK8   -------------------------------------------------PGLMVLR-DPTG--RDLGARYELGGELGRGEFGITYLCTEA---ETGDR---YACKSISKRK---------------------------LRTPVDVED  108
TaCPK12  -------------------------------------------------AGLVVLR-DPTG--RDLDDKYVLGGELGRGEFGVTYLCTDA---ATGAR---MACKSISKRK---------------------------LRTPVDVED  104
OsCPK20  -------------------------------------------------HRLVVLR-EPTG--RDIAARYELGGELGRGEFGVTYLCTER---ETGDA---YACKSISKKK---------------------------LRTAVDIED  119
TaCPK7   -------------------------------------------------SRLVVLR-EPTGTGRDIAERYELGGELGRGEFGVTYLCTDR---ATREA---LACKSISKKK---------------------------LRTAVDVED  112
OsCPK9   -------------------------------------------------AVRVLEGVVPHHPRLRVTDKYQLGRELGRGEFGVTHLATDR---ATRER---LACKSIPKRR---------------------------LRTAVDVAD  145
TaCPK19  --------------------------------------------------ARVLDGVVPHHPRLRVTDKYHLGRELGRGEFGVTRLATDRG--AARER---LACKSIPKAR---------------------------LRTAVDVAD  140
OsCPK21  ---------------------------------------------DGGDVVAIA-----TTTADEFARRYVLGKELGRGEFGVTRRCSDA---ATGEA---LACKTIRKHRRLAP------------------PRVTAAKAAAHGED  131
OsCPK22  ---------------------------------------------GGGRKNAILGDAADVKTAAGFAERYRLGAELGRGEFGVTRRCSDA---ATGEA---LACKTIRRKR--------------------------LRRCRGDAED  150
OsCPK29  ---------------------------------------------NGG-----------VMAAEEMLRRYEIGEELGRGEFGVTRRCRDA---VTGER---LACKSISKRK---------------------------LRSSVDVED  128
OsCPK1   ----------------------------------------------AAPDAAMGRVLGRPM--EDVRATYTFGRELGRGQFGVTYLVTHK---ATGKR---FACKSIATRK---------------------------LAHRDDIED  110
TaCPK8   ----------------------------------------------EAGQVAMGRVLGRPM--EDVRATYTFGRELGRGQFGVTYLVTHK---ATGQR---FACKSIATRK---------------------------LVHRDDIED  110
OsCPK15  ----------------------------------------------AAAEDGVGRVLGRPM--EDVRATYTFGRELGRGQFGVTYLATHK---PTGRR---YACKSIAARK---------------------------LARPDDLDD  134
OsCPK2   ----------------------------------------------NKPPAPIGPVLGRPM--EDVRSIYTIGKELGRGQFGVTSLCTHK---ATGQK---FACKTIAKRK---------------------------LSTKEDVED  105
TaCPK13  ----------------------------------------------NKPPAAIGPVLGRPM--EDVRSIYTVXKELGRGQFGVTSLCTHK---ATGQK---XACKTIAKRK---------------------------LSTKEDVED  107
OsCPK14  ----------------------------------------------AKPPAPIGPVLGRPM--EDVKSIYTVGKELGRGQFGVTSLCTHK---ATGQR---FACKTISKRK---------------------------LSTKEDVED  112
OsCPK25  ----------------------------------------------KKPAGPIGEVLERPM--EEVRTTYSIGKELGRGQFGVTHLCTHK---ATGEK---LACKTIAKRK---------------------------LANKEDVDD  127
OsCPK26  ----------------------------------------------KKPAGPIGEVLERPM--EEVRTTYSIGKELGRGQFGVTHLCTHK---ATGEK---LACKTIAKRK---------------------------LANKEDVDD  127
OsCPK12  ----------------------------------------------SGGGGEMGPVLQRAM--VSVRSLYQLDRKLGSGQFGTTYLCTER---ATGNR---YACKSVSKRK---------------------------LVRRTDVDD  135
TaCPK18  ----------------------------------------------PVG--EIGPVLQRPM--ADVRALYNLERKLGSGQFGTTYLCTER---ATGLK---YACKSVSKRK---------------------------LVRRADVED  138
OsCPK19  ----------------------------------------------APPKPTPDTILGKPY--DDVRSVYYLGKELGRGQFGVTYLCTEI---ASGKQ---YACKSISKRK---------------------------LVSKADKED  129
TaCPK5   ----------------------------------------------APSKPMPDTILGKQY--EDVRSVYSLGKELGRGQFGVTYLCTEI---STGRQ---YACKSISKRK---------------------------LVSKADKED  129
ZmCDPK2  ----------------------------------------------VPPKPTADTILGKQY--EDVRSVYSFGKELGRGQFGVTYLCTEI---ASGRQ---YACKSISKRK---------------------------LVSKADRED  109
ZmCDPK9  ----------------------------------------------VPPKPTADTILGKQY--EDVRSVYSLGKELGRGQFGVTYLCTEV---ASGKQ---YACKSISKRK---------------------------LTSKADRED  127
MtCDPK3  ----------------------------------------------KQD---SNTILGKGL--EDVKQFYTLGKELGRGQFGVTFLCTEN---STGLL---YACKSISKRK---------------------------LVSKSDRED  128
MsCDPK3  ----------------------------------------------KQD---TNTILGKGL--EDVKQFYTLGKELGRGQFGVTFLCTEN---STGLL---YACKSISKRK---------------------------LVSKSDRED  132
OsCPK10  TTDTASAEQDASKSSAGSDSGEAARPRPRV----------PPVKRVSSAGLLVGSVLKRKT--ESLKDKYSLGRKLGQGQFGTTYLCVER---ATGKE---FACKSILKRK---------------------------LVTDDDVED  177
ZmCPK10  ASHSEPAPMAAKPGGAAANASPSPSPRPR-----------PQVKRVSSAGLLLGSVLRRKT--ENLKDKYSLGRRLGQGQFGTTHLCVER---ATGKE---LACKSILKRK---------------------------LGSDDDVED  197
OsCPK17  IADSDLSSS--TPNKGGNK---------------------PKVRRVQSAGLLADSVLKRDS--ERLKDLYTLGKKLGQGQFGTTYQCVEK---ATGKV---LACKSIAKRK---------------------------LVSEEDVED  150
TaCPK9   LDDSDLSS----PSSDPNK---------------------PKVKRVQSAGLIAESVLKRDS--ERIKDLYTLGKKLGQGQFGTTYKCVEK---ATGKE---FACKSIAKRK---------------------------LVTEEDVKD  144
OsCPK11  SHHSSRSTDPSTPTSAAEQPANKAA---------------PKVKRVQSAGLLADSVLKRDVNTARLKDLYTIGKKLGQGQFGTTYLCVEK---ATGRE---FACKSIAKRK---------------------------LLTQEDVED  157
OsCPK27  SQPPPPQEQPSPPPPASSNTTQQPPPPQQRQQSRAKKPAHIKRISSAGLQVESVLRRKT--ENLKDKYSLGRKLGQGQFGTTYLCVDK---ANGGE---YACKSIAKRK---------------------------LLTDEDVED  194
OsCPK24  ----------------------------------------PPP-VTAAG-GRPVSVLPHKT--ANVRDHYRIGKKLGQGQFGTTYLCVDK---ASGGE---FACKSIPKRK---------------------------LLCREDYED   91
TaCPK4   ----------------------------------------PVAXAPAPS-GRPASVLPHKT--ANVRDHYRIGKKLGQGQFGTTYLCVAK---EDGGE---FACKSIPKRK---------------------------LLCHEDYED   92
ZmCPK11  ----------------------------------------QLVTAPAPSSGRPASVLPYKT--ANVRDHYRIGKKLGQGQFGTTYQCVGK---ADGAE---YACKSIPKRK---------------------------LLCREDYED   88
OsCPK28  ----------------------------------------PPVTAPSVG--RPASVLPHKT--ANVRDHYRIGKKLGQGQFGTTYLCVGK---PDGGE---YACKSIPKRK---------------------------LLCREDYED   93
OsCPK13  ---------------------------------------------QAPADLGS--VLGHPT--PNLRDLYAMGRKLGQGQFGTTYLCTEL---STGVD---YACKSISKRK---------------------------LITKEDIED  132
TaCPK2   --------------------------------------------AGAPADLGS--VLGHPT--PNLRDLYALGRKLGQGQFGTTYLCTEL---ATGAD---YACKSISKRK---------------------------LITKEDIDD  139
ZmCDPK1  --------------------------------------------AGAPPDLGS--VLGHTT--PNLRDLYALGRKLGQGQFGTTYLCTEL---ATGID---YACKSISKRK---------------------------LITKEDVDD   71
ZmCDPK7  --------------------------------------------AGAPPDLGS--VLGHPT--PNLRDLYALGRKLGQGQFGTTFLCTEL---ATGVD---YACKSISKRK---------------------------LITREDVDD  135
OsCPK5   --------------------------------------------APAPAELTAN-VLGHPT--PSLSEHYALGRKLGQGQFGTTYLCTDL---ATGVD---YACKSIAKRK---------------------------LITKEDVED  136
OsCPK7   ------------------------------------------------LDPTSINVLGRKT--ADLREHYIIGRKLGQGQFGTTYLCTEI---NTGCE---YACKTIPKRK---------------------------LITKEDVED  123
TaCPK1   ------------------------------------------------LDLTSISVLGRKT--ADLREHYIIGRKLGQGQFGTTYLCTEI---STGCD---FACKTILKRK---------------------------LVTKVDVED  125
OsCPK23  ------------------------------------------------LNLKSP-VLGYKT--PNVRELYTLGRELGQGQFGKTYLCTEI---STGCQ---YACKTILKSN---------------------------LRCVSDIED  117
OsCPK6   ---------------------------------------------------SPTAVLGHET--PALREVYTVGRKLGQGQFGTTYLCTQV---STGAE---YACKSIAKRK---------------------------LLSPEDVED  118
OsCPK31  ----------------------------------------------ARTELDPEEIKSVFGYPRNLLDSYYLGRVIGAGSFGVVREGIEV---STGRR---FAVKTVSKVPKR------------------------GSPTPRYLLK  146
OsCCaMK  ---------------------------------------------------------MSKTESRKLSDDYEVVDVLGRGGFSIVRRGVSK----SEE-KTQVAIKTLRRLGPAMA--GMKQGT-KPVPGSG-LPMWKQVSISDAL   79
TaCCaMK  ---------------------------------------------------------MSTTESRRLSDDYEVADVLGRGGFSIVRRGVSK----SDEGKTQVAIKTLRRLGPAMTGMGMQQGS-KDAPSSGGLPLWKQVSISDAL   83
MtCCaMK  ---------------------------------------------------------MGYG-TRKLSDEYEVSEILGRGGFSVVRKGTKKSSIEEEKSQSQVAIKTLRRLGASNNPSGLPRKKDIGEKSTIGFPTMRQVSVSDTL   87
```

```
OsCPK18   VKREVKILKALQ--------GHEN---VVHFYNAFEDDNY--VYIVMELCEGGELLDRILAKKDSRYSEKDAAVVVRQMLKVAAECHLHGLVHRDMKPENFLFKSTKEDSSLKATDFGLSDFIRPGK-HFRDIVGSAYYVAPE   225
TaCPK6    VKREVKILKALH--------GHEN---VVHFYNAFEDDNY--IYIVMELCEGGELLDHILAKKDSRYSEKDAAVVVRQMLKVAAECHLHGLVHRDMKPENFLFKSSKEGSPLKATDFGLSDFITPGK-QFRDIVGSAYYVAPE   224
MtCDPK1   VKREVKILKELT--------GHEN---VVQFYNAFDDDSY--VYIVMELCEGGELLDRILNKKDSRYTEKDAAVVVRQMLKVAAQCHLHGLVHRDMKPENFLFKSNKEDSALKATDFGLSDFIKPGK-RFQDIVGSAYYVAPE   272
OsCPK4    VKREVKILKELK--------GHEN---IVHFYNAFEDDSY--VYIVMELCEGGELLDRILAKKNSRYSEKDAAVVVRQMLKVAAECHLHGLVHRDMKPENFLFKSTKEDSPLKATDFGLSDFIKPGK-KFHDIVGSAYYVAPE   232
OsCPK30   VRREVRILSSLT--------GHSN---LVQFYDAFEDEDN--VYIVMELCKGGELLDRILAR-GGKYSEEDAKVVMVQILSVVSFCHLQGVVHRDLKPENFLFTSKDENSALKVIDFGLSDFVKPDE-RLNDIVGSAYYVAPE   316
OsCPK3    VRREVAIMRHLP--------KSAS---IVSLREACEDEGA--VHLVMELCEGGELFDRIVAR--GHYTERAAANVTRTIVEVVQLCHRHGVIHRDLKPENFLFANKKENSPLKAIDFGLSIFFKPGE-KFSEIVGSPYYMAPE   248
TaCPK3    VRREVAIMRHLP--------RSHS---IVALREACEDEGA--VHLVMELCEGGELFDRIVAR--GQYTERAAANVTRTIVEVVQLCHRHGVIHRDLKPENFLFANKKENSPLKAIDFGLSIFFKPGE-KFSEIVGSPYYMAPE   242
OsCPK16   VRREVAIMRHLP--------RSAS---IVSLREACEDDGA--VHLVMELCEGGELFDRIVAR--GHYTERAAAAVTRTIVEVVQLCHRHGVIHRDLKPENFLFANKKENSPLKAIDFGLSIFFKPGE-KFSEIVGSPYYMAPE   244
TaCPK15   VRREVAIMRHLP--------RSAS---IVTLREACEDDGA--GHLVMELCEGGELFDRIVAR--GHYTERAAAAVTRTIVEVVQLCHHHGVIHRDLKPENFLFANKKENSPLKAIDFGLSIFFKPGE-KFSEIVGSPYYMAPE   248
OsCPK8    VRREVEIMRHMP--------SHPN---IVSLRAAYEDEDN--VHLVMELCEGGELFDRIVAR--GHYTERAAAAVTRTIVEVVQMCHRHGVMHRDLKPENFLYANKKDSSPLKAIDFGLSVFFRPGE-RFTEIVGSPYYMAPE   235
TaCPK12   VRREVEIMRHTP--------PHPN---IVSLSAAYEDEDA--AHLVMELCEGGELFDRIVAR--GHYTERAAAAVTRTIAEVVQMCHRNGVIHRDLKPENFLYANKKESSPLKAIDFGLSVFFRPGE-RFSEIVGSPYYMAPE   231
OsCPK20   VRREVDIMRQLP--------KHPN---IVTLRDTYEDDNA--VHLVMELCEGGELFDRIVAR--GHYTERAAALVTRTIVEVVQMCHKHGVMHRDLKPENFLFANKKETAALKAIDFGLSVFFTPGE-RFTEIVGSPYYMAPE   246
TaCPK7    VRREVEIMRHLP--------KHPN---IVTLRDTYEDDNA--VHLVMELCEGGELFDRIVAR--GHYTERAAAVVTKTIVEVVQMCHKHGVMHRDLKPENFLFANKKETAVLKAIDFGLSVFFTPGE-RFTEIVGSPYYMAPE   239
OsCPK9    VRREVAIMASLP--------DHPA---LVRLRAAYEDADA--VHLVMELCDGGELFDRIVAR--GRYTERAAAAARTVAEVVRACHAHGVMHRDLKPENFLYAGKAEDAQLKAIDFGLSVFFRPGE-RFREIVGSPYYMAPE   272
TaCPK19   VRREVAIMASLP--------DHPA---LVRLRAAYEDDEA--VHLVMELCDGGELFDRIVAR--GRYTERAAAAARTVAEVVRACHAHGVMHRDLKPENFLYAGKEEDAQLKAIDFGLSVFFRPGE-RFSEIVGSPYYMAPE   267
OsCPK21   VKREVAIMRRMSSASSSRGGGAASSAAVVRLREACEDAADGSVHLVMELCEGGELFDRIVAR--GHYSERAAANIFRTIVDVVQLCHSNGVIHRDLKPENFLFANKSEDSPLKVIDFGLSVFFKPGD-RFTEVVGSAYYMAPE   271
OsCPK22   VRREVEILRRIS----ALGAGADS---VVRLRDACEDSDG--VHLVMELCEGGELFDRIFAR--GHYTERAAAKLARTIVGVVQLCHENGVMHRDLKPENFLFANKSEDSPLKAIDFGLSVFFKPGE-RFTQVVGSTYYMAPE   281
OsCPK29   VRREVAIMRSLP--------AHAN---VVRLREAFEDADA--VHLVMEVCEGGELFDRIVAR--GHYTERAAAAVMRTIMDVVQHCHKNGVMHRDLKPENFLYANASENSPLKVIDFGLSVCFKPGA-RFNEIVGSPYYMAPE   255
OsCPK1    VRREVQIMHHLT--------GHRN---IVELRGAYEDRHS--VNLIMELCEGGELFDRIIAR--GHYSERAAAALCREIVAVVHSCHSMGVFHRDLKPENFLFLSKSEDSPLKATDFGLSVFFKPGE-HFKDLVGSAYYVAPE   237
TaCPK8    VQREVQIMHHLT--------GHRN---IVELRGAYEDRHS--VNLIMELCEGGELFDRIIAR--GHYSERAAALLCREMVSVVHSCHSMGVFHRDLKPENFLFLNNKEDSPLKATDFGLSVFFKHGE-QFKDLVGSAYYVAPE   237
OsCPK15   VRREVHIMHHLT--------GHRN---IVELRGAYEDRHS--VNLVMELCEGGELFDRIIAR--GHYSERAAAALCREIVSVVHSCHSMGVMHRDLKPENFLFLNKREDSPLKATDFGLSVFFKPGE-QFRDLVGSAYYVAPE   261
OsCPK2    VRREVQIMYHLA--------GQPN---VVELKGAYEDKQS--VHLVMELCAGGELFDRIIAK--GHYTERAASSLLRTIVEIIHTCHSLGVIHRDLKPENFLLLSKDEDAPLKATDFGLSVFFKQGE-VFKDIVGSAYYIAPE   232
TaCPK13   VRREVQIMYHLA--------GQPN---IVELKGAYEDKLS--VHLVMELCAGGELSDRIIAK--GKYTERAASSLLRTIVEIIHTCHSLGVIHRDLKPENFLLLSKEEDAPLKATVFGLSVLYKQGE-VFKDIVGSAYYIAPE   234
OsCPK14   VRREVQIMYHLA--------GQPG---VVELKGAYEDKHA--VHLVMELCAGGELFDRIIAK--GHYTEHAASSLLRTIVEIIHTCHSMGVIHRDLKPENFLLLSKDEHAPLKATDFGLSVFFKEGE-VFRDIVGSAYYIAPE   239
OsCPK25   VRREVQIMHHLS--------GQPN---IVDLRGAYEDKHN--VHLVMELCAGGELFDRIIAR--GHYTERAAAALLRAIVGIVHTCHSMGVIHRDLKPENFLLLSKGDDAPLKATDFGLSVFFKEGE-VFRDIVGSAYYIAPE   254
OsCPK26   VRREVQIMHHLS--------GQPN---IVDLRGAYEDKHN--VHLVMELCAGGELFDRIIAR--GHYTERAAAALLRAIVGIVHTCHSMGVIHRDLKPENFLLLSKGDDAPLKATDFGLSVFFKEGE-VFRDIVGSAYYIAPE   254
OsCPK12   VRREITILQHLS--------GQPN---IAEFRGAYEDNDH--VHLVMEFCSGGELFDRITAK--GSYSERQAAAVCRDILTVVHVCHFMGVIHRDLKPENFLLASADDDAPLKAIDFGLSVFIEEGK-VYKDIVGSAYYVAPE   262
TaCPK18   MRREVTILQHLS--------GQPN---IAEFRGAFXDAES--VHLVMEFCSGGELFDRITAK--GSYSERQAAAVCRDILTVVHVCHFMGVLHRDLKPENFLLASPADDAPLKAIDFGLSVFIEEGK-VYKDIVGSAYYVAPE   265
OsCPK19   IRREIQIMQHLS--------GQQN---IVEFRGAYEDKSN--VHVVMELCAGGELFDRIIAK--GHYSERAAATICRAVVNVVNICHFMGVMHRDLKPENFLLATKEENAMLKATDFGLSVFIEEGK-MYRDIVGSAYYVAPE   256
TaCPK5    IRREIQIMQHLS--------GQPN---IVEFCGAYEDKGS--VHVVMELCAGGELFDRIIAK--GHYSERAAATICRGVVNVVNVCHFMGVMHRDLKPENFLLATKDENAVLKATDFGLSVFIEEGK-MYRDIVGSAYYVAPE   256
ZmCDPK2   IRREIQIMQHLS--------GQPN---IVEFRGAYEDKSN--VHVVMELCAGGELFDRIIAK--GHYTERAAATICRAVVNVVNICHFMGVMHRDLKPENFLLATMEENAMLKATDFGLSVFIEEGK-MYRDIVGSAYYVAPE   236
ZmCDPK9   IRREIQIMQHLS--------GQPN---IVEFRGAYEDKSN--VHVVMELCAGGELFDRIIAK--GHYTERAAATICRAVVNVVNICHFMGVMHRDLKPENFLLASKEENAMLKATDFGLSVFIEEGK-MYRDIVGSAYYVAPE   254
MtCDPK3   IKREIQIMQHLS--------GQPN---IVEFKGAYEDRNS--VHVVMELCAGGELFDRIIAK--GHYSEKAASICRQIVNVVNICHFMGVMHRDLKPENFLLASKDENALLKATDFGLAVFIEEGK-VYRDIVGSAYYVAPE   255
MsCDPK3   IKREIQIMQHLS--------GQPN---IVEFKGAYEDRNS--VHVVMELCAGGELFDRIIAK--GHYSEKAASICRQIVNVVNICHFMGVMHRDLKPENFLLASKDENALLKATDFGLSVFIEEGR-VYRDIVGSAYYVAPE   259
OsCPK10   VRREIQIMYHLA--------GHPN---VISIRGAYEDAVA--VHLVMELCAGGELFDRIVQK--GHYTERKAAELARVIVGVVEVCHSMGVMHRDLKPENFLFADQTEEAALKTIDFGLSIFFRPGQ-VFTDVVGSPYYVAPE   304
ZmCPK10   VRREIQIMHHLA--------GHPS---VVGIRGAYEDAVA--VHLVMELCGGGELFDRIVRR--GHYTERKAAELARVIVGVVEACHSMGVMHRDLKPENFLFADHSEEAALKTIDFGLSIFFRPGQ-IFTDVVGSPYYVAPE   324
OsCPK17   VRREIQIMHHLA--------GHPS---VVSIVGAYEDAVA--VHLVMELCAGGELFDRIVQR--GHYSEKAAAQLARVIIGVVEACHSLGVMHRDLKPENFLFVNHKEDSPLKTIDFGLSIFFKPGE-NYSDVVGSPYYVAPE   277
TaCPK9    VRREIQIMHHLA--------GHPN---VISIVGAYEDAVA--VHLVMELCAGGELFDRIIQR--GHYSEKAAAQLARVIIGIVEACHSLGVMHRDLKPENFLFVNQKEDSPLKTIDFGLSIFFKPGG-IYSDVVGSPYYVAPE   271
OsCPK11   VRREIQIMHHLA--------GHAN---VVSIVGAYEDAVA--VQLVMELCAGGELFDRIIQR--GHYSEKAAAQLARVIVGVIEACHSLGVMHRDLKPENFLFIHQKEDSPLKAIDFGLSIFFKPGE-TFTDVVGSPYYVAPE   284
OsCPK27   VRREIQIMHHLA--------GHPN---IISIRGAYEDAVA--VHVVMELCAGGELFDRIVRK--GHYTERQAAGLARVIVAVVESCHSLGVMHRDLKPENFLFVGNEEDAPLKTIDFGLSMFFRPGE-VFTDVVGSPYYVAPE   321
OsCPK24   VWREIQIMHHLS--------EHPN---VVRIRGAYEDALF--VHIVMELCAGGELFDRIVAK--GHYTERAAAQLIRTIVAVVEGCHSLGVMHRDLKPENFLFASAAEDAPLKATDFGLSMFYKPGD-KFSDVVGSPYYVAPE   218
TaCPK4    VWREIQIMHHLS--------EHPN---VVRIRGAYEDALF--VHIVMELCAGGELFDRIVAK--GHYTERAAAQLIRTIVGVIEACHSLGVMHRDLKPENFLLASTAEDAPLKTTDFGLSMFYKPGD-KFSDVVGSPYYVAPE   219
ZmCPK11   VYREIQIMHHLS--------EHPN---VVRIRGAYEDALF--VHIVMELCAGGELFDRIVAK--GHYSERAAAKLIKTIVGVVEGCHSLGVMHRDLKPENFLFASTAEEAPLKATDFGLSMFYKPGD-KFSDVVGSPYYVAPE   215
OsCPK28   VWREIQIMHHLS--------EHPN---VVRIRGAYEDALF--VHIVMELCAGGELFDRIVAK--GHYTERAAALLIRTIVGVVEGCHSLGVMHRDLKPENFLFASTAEDAPLKATDFGLSVFYKPGD-KFSDVVGSPYYVAPE   220
OsCPK13   VRREIQIMHHLS--------GHKN---VVAIKGAYEDQLY--VHIVMELCAGGELFDRIIQR--GHYSERKAAELTRIIVGVVEACHSLGVMHRDLKPENFLLANKDDDLSLKAIDFGLSVFFKPGQ-TFTDVVGSPYYVAPE   259
TaCPK2    VRREIQIMHHLS--------GHRN---VVAIKGAYEDQLY--VHIVMELCAGGELFDRIIQR--GHYSERKAAELTRIIVGVVEACHSLGVMHRDLKPENFLLANKDDDLSLKAIDFGLSVFFKPGQ-IFTDVVGSPYYVAPE   266
ZmCDPK1   VRREIQIMHHLS--------GHKN---VVAIKGAYEDQVY--VHIVMELCAGGELFDRIIQR--GHYSERKAAELTRIIVGVVEACHSLGVMHRDLKPENFLLANRDDDLSLKAIDFGLSVFFKPGQ-VFTDVVGSPYYVAPE   198
ZmCDPK7   VRREIQIMHHLS--------GHTN---VVAIKGAYEDQLY--VHIVMELCAGGELFDRIIQR--GHYSERKAAELTRIIVGVVEACHSLGVMHRDLKPENFLLVNKDDDLSLKAIDFGLSVFFKPGQ-VFTDVVGSPYYVAPE   262
OsCPK5    VRREIQIMHHLA--------GHRN---VVAIKGAYEDPQY--VHIVMELCAGGELFDRIIER--GQFSERKAAELTRIIVGVIEACHSLGVIHRDLKPENFLLANKDDDLSLKAIDFGLSVFFKPGQ-VFTDVVGSPYYVAPE   263
OsCPK7    VRREIQIMHHLS--------GHKN---VVAIKDVYEDGQA--VHIVMELCAGGELFDRIQEK--GHYSERKAAELIRIIVSIVAMCHSLGVMHRDLKPENFLLLDKDDDLSIKAIDFGLSVFFKPGQ-AFTELVGSPYYVAPE   250
TaCPK1    VRREIQIMHHLS--------GHKN---VVSIKDVYEDVQA--VHIVMELLPGGELFDRIQGN--GHYSEMKAAEITRIIVSIVAMCHSLGVMHRDLKPENFLLLDKDDDLSIKAIDFGLSVYFKPGQ-VFSELVGSPFYVAPE   252
OsCPK23   VRREIQIMHHLS--------GQKN---IVTIKDTYEDEQA--VHIVMELCAGGELFSKIQKR--GHYSERKAAELIKIIVGIIETCHSHGVMHRDLKPENFLLLDADDEFSVKAIDFGLSVFFKPGQ-VFREVVGSAYYIAPE   244
OsCPK6    VRREIQIMHHLA--------GHGS---VVTIQGAYEDNLY--VHIVMELCEGGELFDRIVER--GYFSERKAAEITRVIVGVVEACHSLGVMHRDLKPENFLLKESSSSSSLKAIDFGLSVFFKPGQ-VFSDVVGSPYYVAPE   245
OsCPK31   LRAEVEVMQQLG--------VSLN---AVHLHDVFEDDVN--VHMVMELCEGGALLERVESG---VYSELYISKLVRSILRFIAQCHAKGIIYRDVNPDNFLFLTAEEDSPLKATDFGLSIRHYSHEPKLTSRSGTPAYMAPE   273
OsCCaMK   LTNEILVMRRIVES----VAPHPN---VINLHDVYEDVHG--VHLVLELCSGGELFDRIVGR--DRYSEFDAACVIRQIASGLEALHKASIVHRDLKPENCLFSDKDEKSTLKIMDFGLSSVEDFSD-PIVALFGSIDYVSPE   210
TaCCaMK   LTNEILVMRRIVEN----VAPHPN---VISLHDVYEDAHG--VHLVLELCSGGELFDRIIGR--ERYSEFDAAAVVRQIAGGLKALHKANIIHRDLKPENCLFSDRKEDSTLKIMDFGLSSVEDFSD-PVVALFGSVDYVSPE   214
MtCCaMK   LTNEILVMRRIVEN----VSPHPN---VIDLYDVYEDTNG--VHLVLELCSGGELFDRIVAQ--DKYSETEAATVVHQIASGLEAVHRANIVHRDLKPENCLFLDVRKDSPLKIMDFGLSSVEEFTD-PVVGLFGSIDYVSPE   218
```

```
OsCPK18   VLKR-KSGPESDVWSIGVITYILLCGRRPFWD------KTEDGIFKEVLKNKPDFRRKPWPNITPCAKDFVQKLLVKDPRARLTAAQALSHEWVREGGQASDIPLDISVLHNMRQFVKYSRFKQFALRALAST----------  351
TaCPK6    VLKR-KSGPESDVWSIGVITYILLCGRRPFWD------KTEDGIFKEVLKNKPDFRRKPWANITPSAKDFVQKLLVKVPRARLTAAQALSHEWVREGGQASEIPLDISVLHNMRQFVKYSRFKQFALRALAST----------  350
MtCDPK1   VLKR-KSGPESDVWSIGVITYILLCGRRPFWD------KTEDGIFKEVLRNKPDFRRKPWPTISNAAKDFVKKLLVKDPRARLTAAQALSHPWVREGGEASEIPIDISVLNNMRQFVKYSRLKQFALRALAST----------  398
OsCPK4    VLKR-RSGPESDVWSIGVITYILLCGRRPFWN------KTEDGIFREVLRNKPDFRKKPWPGISSGAKDFVKKLLVKNPRARLTAAQALSHPWVREGGEASEIPVDISVLSNMRQFVKYSRFKQFALRALAST----------  358
OsCPK30   VLHR-SYGTEADMWSIGVIAYILLCGSRPFWA------RTESGIFRAVLKAEPSFDEAPWPTLTAEAKDFVKRLLNKDYRKRMTAAQALSHPWIRNS-QQVKIPLDMIIYKLMRAYISSSSLRKSALRALAKT----------  441
OsCPK3    VLKR-NYGPEIDIWSAGVILYILLCGVPPFWA------ETEQGVAQAILRGNIDFKREPWPNVSENAKDLVRRMLEPDPKLRLTAKQVLEHPWLQNAKKAPNVPLGDIVKSRLKQFSRMNRFKRRALRVIADH----------  374
TaCPK3    VLKR-NYGPEIDIWSAGVILYILLCGVPPFWA------ETEQGVAQAILRGNIDFKREPWPNVSENAKDLVRRMLEPDPKLRLTAKQVLEHPWLQNAKKAPNVPLGDIVKSRLKQFSRMNRFKRRALRVIADH----------  368
OsCPK16   VLKR-NYGPEIDIWSAGVILYILLCGVPPFWA------ETEQGVAQAILRGNIDFKREPWPNVSDNAKDLVRQMLQPDPKLRLTAKQVLEHTWLQNAKKAPNVPLGDIVKSRLKQFSRMNRFKRRALRVIADH----------  370
TaCPK15   VLKR-NYGPEIDIWSAGVILYILLCGVPPFWA------ETEQGVAQAILRGNIDFKREPWPHVSDNAKDLDRQMLQPDPKIRLTAKQVLEHTWLQNAKKAPNVPLGDIVKSRLKQFSRMNRFKRRALRVIADH----------  374
OsCPK8    VLKR-HYGPEVDVWSAGVILYILLCGVPPFWA------ETEQGVAQAIIRSVVDFKREPWPRVSEPAKDLVKRMLDPNPMTRLTAEQVLEHPWLHDSKKMPDIPLGDAVRARLQQFAAMNKLKKKALKVIAEH----------  361
TaCPK12   VLKR-NYGPEIDVWSAGVILYILLCGVPPFWA------ETEQGVAQAIIRSVVDFKRDPWPRVSEPAKDLVRRMLDPNPIDGLTAAQVLEHPWLHDSKKNPDIQLGDTVRARLQQFSAMNKLKKKALRVIAEH----------  357
OsCPK20   VLKR-NYGPEVDVWSAGVILYILLCGVPPFWA------ETEQGVAQAIIRSVIDFKRDPWPRVSDNAKDLVKGMLNPDPRRRLNAQQVLDHPWLQNIKKAPNVNLGETVKARLQQFSVMNKFKKHALRVIAEH----------  372
TaCPK7    VLKR-NYGQEVDVWSAGVILYVLLCGVPPFWA------ETEQGVAQAIIRSGIDFKRDPWPRVSDNAKDLVRGMLNPDPRRRLTAQQVLDHPWLQSIKRAPNVNLGETVKARLQQFSVMNKFKKHALRVIAEH----------  365
OsCPK9    VLRR-DYGPEVDIWSAGVILYILLCGVPPFWA------ETEQGVARAILRGAADFDREPWPRISRAAKSLVRQMLDVDPRRRPTAQQVLDHPWLHHAARAPNVPLGDVVRARLKQFSLMNRLKKKAMRVIAEH----------  398
TaCPK19   VLRR-SYGPEVDIWSAGVILYILLCGVPPFWA------ETEQGVARSILRGVLNFDREPWPRISDSAKSLVRQMLEMDPRKRLTARQVLAHPWLQDAKKAPNVPLGDVVRARLKQFSVMNRFKKKAMRVIAEH----------  393
OsCPK21   VLRR-SYGPEVDVWSAGVILYILLCGVPPFWG------DNDEKIAQAILRGAIDFNREPLPRVSANAKDLVRRMLDPNPSTRLTAKQVLEHPWLKNADTAPNVSLGDAVRARLQQFSAMNKFKKKALGVVARN----------  397
OsCPK22   VLNR-SYGPEADVWSAGVILYILLCGVPPFWG------DNDEKTVTAILQGGINFQREPWPKVSPHAKDLVSKMLDPDPSTRLTAKEVLEHPWLKNADRAPNVSLGEIVRSRLMQFSAMNKFKKKALGVVAKN----------  407
OsCPK29   VLKR-NYGQEIDIWSAGVILYILLCGVPPFWA------ETDEGIAQAIIRSHIDFQREPWPKVSDNAKDLVRRMLDPNPYTRLTAQQVLEHPWIQNASAAPNIPLGEAVRSRLKQFTVMNKFKKKALLVVAEY----------  381
OsCPK1    VLKR-NYGAEADIWSAGVILYILLSGVPPFWA------ESEDGIFDAVLRGHIDFSSEPWPSISNGAKDLVKKMLRQDPKERLTSAEILNHPWIREDGEAPDKPLDITVISRMKQFRAMNKLKKVALKVVAEN----------  363
TaCPK8    VLKR-HYGAEADIWSAGIILYILLSGVPPFWA------DNEDGIFEAVLLGHIDFSSDPWPSISNGAKDLVKKMLRQDPKERLTAAEILNHPWIREDGEAPDKPLDITVISRMKQFRAMNKLKKVALKVIAEN----------  363
OsCPK15   VLKR-LYGAEADIWSAGVILYILLSGVPPFWA------ENEDGIFDAVLQGHIDFSSEPWPSISSGAKDLVKRMLRQDPKERLTAAEILNHPWIREDGEAPDKPLDITVISRMKQFRAMNKLKKVALKVVAEN----------  387
OsCPK2    VLKR-SYGPEADIWSVGVILYILLCGVPPFWA------ESEHCIFNSILRGQVDFTSDPWPRISASAKDLVRKMLNSDPKKRISAYEVLNHPWIKEDGEAPDTPLDNAVMNRLKQFRAMNQFKKAALRVIAGC----------  358
TaCPK13   VPKR-NYGPEADIWSVGVIVYILLCGVPPFWA------ESEHGIFNSILRGQIDFSSGPWPRISPGAKDLVRKMLNSDPKKRISAYDVLNHPWIKEDGEVPDTPLDNAVMNRLKQFKAMNQFKKAALRVIAGC----------  360
OsCPK14   VLKR-SYGPEADIWSIGVMLYILLCGVPPFWAGKLLLFIAMHGIFNSILRGHVDFSSEPWSRISHGAKDLVRRMLHSDPKQRISAYDVLNHPWIKEDGEAPDTPLDNAVLGRLKQFRAMNQFKKAALRVIAGC----------  371
OsCPK25   VLKR-KYGPEADIWSIGVMLYIFLAGVPPFWA------ESENAIFTAILRGQIDLASEPWPKISSGAKDLVRKMLNINPKERLTAFQVLNHPWIKEDGDAPDVPLDNVVLNRLKQFRAMNQFKKAALRIIAGC----------  380
OsCPK26   VLKR-KYGPEADIWSIGVMLYIFLAGVPPFWA------ESENAIFAAILRGQIDLASEPWPKISSGAKDLVRKMLNINPKERLTAFQVLNHPWIKEDGDAPDVPLDNVVLNRLKQFRAMNQFKKAALRIIAGC----------  380
OsCPK12   VLQR-NYGKEADIWSAGVILYILLCGTPPFWA------ETEKGIFDAILVNQVDFSTSPWPSISESAKDLIRQMLHRDPQKRITASQALEHRWLKEG-GASDRPIDSAVLSRMKQFKAMNKLKQLALKVIAEN----------  387
TaCPK18   VLHR-NYGREIDVWSAGVILYILLCGSPPFWA------ETEKGIFDAILVGQLDFSSSPWPTISESAKDLIRQMLNRDPKRRITAAQALEHPWLKEG-GASDRPIDSAVLSRMKQFKAMNKLKQLALKVIAEN----------  390
OsCPK19   VLRR-NYGKEIDVWSAGVILYILLSGVPPFWA------ETEKGIFDAILQGEIDFESQPWPSISESAKDLVRKMLTQDPKKRITSAQVLQHPWLRDG-EASDKPIDSAVLSRMKQFRAMNKLKKMALKVIASN----------  381
TaCPK5    VLRR-NYGKEIDVWSAGVILYILLSGVPPFWA------ETEKGIFDAILQGEIDFESQPWPSISESAKDLVRKMLAQDPKKRISSAQVLQHPWLREG-EASDKPIDSAVLSRMKQFRAMNKLKKMALKVIASN----------  381
ZmCDPK2   VLRR-SYGKEIDVWSAGVILYILLSGVPPFWA------EIEKGIFDAILHEEIDFESQPWPSISESAKDLVRKMLTRDPKKRLTSAQVLQHQWLREGGEASDKPIDSAVLSRMKQFRAMNKLKKMALKVIASN----------  362
ZmCDPK9   VLKR-SYGKEIDVWSAGVILYILLSGVPPFWA------ETEKGIFDAILHEEIDFESQPWPSISESAKDLVRKMLTRDPKKRLTSAQVLQHSWLREGGGASDKPIDSAVLSRMKQFRAMNKLKKMALKVIASN----------  380
MtCDPK3   VLRR-RCGKEIDIWSAGVILYILLSGVPPFWA------ETEKGIFDAILEGHIDFESEPWPKISDSAKDLVRKMLIQEPKKRITAAQVLEHPWIKDG-NASDKPIDSAVLSRMKQFRAMNKLKKLALKVIAEN----------  380
MsCDPK3   VLRP-RCGKEIDIWSAGVILYILLSGVPPFWA------ETEKGIFDAILEGHIDFESEPWPKISDSAKDLVRKMLIQEPKKRITAAQVLEHPWIKGG-NASDKPIDSAVLSRMKQFRAMNKLKKLALKVIAEN----------  384
OsCPK10   VLKK-KYGQEADVWSAGVIIYILLCGVPPFWA------ENEQGIFEEVLHGRLDFQSEPWPSISEGAKDLVRRMLVRDPKKRLTAHEVLRHPWVQVGGLAPDKPLDSAVLSRMKQFSAMNKLKKMALRVIAEN----------  430
ZmCPK10   VLKK-RYGPEADVWSAGVIIYILLCGVPPFWA------ENEQGIFEEVLHGRLDFESEPWPSISDGAKDLVRRMLVRDPRKRLTAHEVLRHPWVQVGGVAPDRPLDSAVLSRMKQFSAMNKLKKMALRVIAEN----------  450
OsCPK17   VLMK-HYGREVDVWSAGVIIYILLSGVPPFWD------ESEQGIFEKVLKGDLDFSSDPWPAISDSAKDLVRKMLNRDPRKRLTAHEALCHPWVCVDGVAPDKPLDSAVLTRLKQFSAMNKLKKMALRVIAEN----------  403
TaCPK9    VLLK-QYGCEVDVWSAGVIIYILLSGVPPFWD------ESEQGIFEQVLQGDLDFSSEPWPSISKSAKDLVRKMLNRDPGKRLTAHEALCHPWVCVDGVAPDKPLDSAVLTRLKQFSAMNKLKKMALRVIAEN----------  397
OsCPK11   VLMK-HYGREVDVWSAGVIIYILLSGVPPFWD------ESEQGIFEQVLKGDLDFSSEPWPNISESAKDLVRKMLIRDPKKRLTAHEALCHPWVCVDGVAPDKPLDSAVLSRLKQFSAMNKLKKMALRVIAES----------  410
OsCPK27   VLKK-SYGQEADVWSAGVIIYILLCGVPPFWA------ETEQGIFEQVLHGTLDFESDPWPNVSDGAKDLLRKVLVRDPKKRLTAHEVLCHPWLQMSGSAPDKPLDSAVLSRLRQFSAMNKLKKMALRVIAEN----------  447
OsCPK24   VLQK-CYGPESDVWSAGVILYILLCGVPPFWA------ETEAGIFRQILRGKLDFESEPWPSISDSAKDLVRNMLCRDPTKRLTAHEVLCHPWIVDDAVAPDKPIDSAVLSRLEHFSAMNKLKKMALRVIAES----------  344
TaCPK4    VLQK-CYGPEADVWSAGVILYILLCGVPPFWA------ETEAGIFRQILRGKLDFESEPWPSISDSAKDLVRTMLCRDPTKRLSAHEVLCHPWIVDDAVAPDKPIDSAVLSRLKHFSAMNKLKKMALRVIAES----------  345
ZmCPK11   VLQK-CYGPEADVWSAGVILYILLCGVPPFWA------ETEAGIFRQILRGKLDFESEPWPSISDSAKDLVCNMLTRDPKKRFSAHEVLCHAWIVDDAVAPDKPIDSAVLSRLKHFSAMNKLKKMALRVIAES----------  341
OsCPK28   VLQK-IYGPEADVWSAGVILYILLCGVPPFWA------ETESGIFRQILRGKLDLESDPWPSISDSAKDLVRNMLIRDPTKRFTAHEVLCHPWIVDDAVAPDKPIDSAVLSRLKHFSAMNKLKKMALRVIAES----------  346
OsCPK13   VLLK-HYGPEADVWTAGVILYILLSGVPPFWA------ETQQGIFDAVLKGFIDFDSDPWPVISESAKDLITKMLNPRPKERLTAHEVLCHPWIRDHGVAPDRPLDPAVLSRIKQFSAMNKLKKMALRVIAES----------  385
TaCPK2    VLCK-KYGPEADVWTAGVILYILLSGVPPFWA------ETQQGIFDAVLKGVIDFDSEPWPVISDSAKDLITSMLNPRPAERLTAHEVLCHPWIRDQGVAPDRPLDTAVLSRIKQFSAMNKLKRMALRVIAES----------  392
ZmCDPK1   VLLK-SYGPAADVWTAGVILYILLSGVPPFWA------ETQQGIFDAVLKGAIDFDSDPWPVISDSAKDLIRRMLNPRPAERLTAHEVLCHPWIRDHGVAPDRPLDPAVLSRIKQFSAMNKLKKMALRVIAES----------  324
ZmCDPK7   VLLK-NYGPAADVWTAGVILYILLSGVPPFWA------ETQQGIFDAVLKGVIDFDSDPWPVISDSAKDLIRRMLNPRSAERLTAHEVLCHPWIRDHGVAPDRPLDPAVLSRIKQFSAMNKLKKMALRVIAES----------  388
OsCPK5    VLRK-CYGPEADVWTAGVILYILLSGVPPFWA------ETQQGIFDAVLKGVIDFDSDPWPVISDSAKDLIRRMLNPRPKERLTAHEVLCHPWICDHGVAPDRPLDPAVLSRIKQFSAMNKLKKMALRVIAES----------  389
OsCPK7    VLHK-RYGPESDVWSAGVILYVLLSGVPPFWA------ETQQGIFDAVLKGHIDFQSDPWPKISDSAKDLIRKMLSHCPSERLKAHEVLRHPWICENGVATDQALDPSVISRLKQFSAMNKLKKLALRVIAER----------  376
TaCPK1    VLHK-RYGPESDVWSAGVILYVLLSGVPPFWA------DTQKGIFDAVLKGHLDLESDPWPKISDGAKDLIRKMLCNCPSERLKAHEVLRHPWICQNGVATDGVLDPSVISRLKRFSAMNNLQKLALRVIAER----------  378
OsCPK23   VLEK-RYGPEADIWTAGVILYVLLTGVPPFWA------DTQSGIYEKVLDGRIDFKSNRWPRISDSAKDLIKKMLCPYPSERLKAHEVLKHPWICDNGVATNRALDPSVLPRLKQFSAMNRLKKLSLQIIAER----------  370
OsCPK6    VLCK-HYGPEADVWTAGVIVYILLSGVPPFWA------ETQQGIFDAVLRGSLDFDSDPWPTISDSAKDLIRRMLRSPPREARLTAHQVLCHPWVCDDGVAPDRPLAPAVLSRLKQFSAMNRLKKMALRVIARN----------  371
OsCPK31   LVMQ-CYDEKADLWSVGMLAYQLLTGRFPFWEDVRN--ETLSDVWKAILSSEIDWNAPELQPLSSAARDLLERLLQRNPVMRPSAAEALEHPWLAQEGAANDMPLKGSVVQRLQRFATYTHLKQVVLRMITED----------  403
OsCCaMK   ALSRQEVSAASDMWSVGVILYILLSGCPPFHAA------TNREKQQRILQGEFSFQDHTWKTISSSAKDLISRLLSVQPYKRPTASDLLRRPWVIGDCAKQDL-MDAEVVSKLQKFNARRKLRAAAIASVLSCKVALRTKRLR  346
TaCCaMK   ALSRQEVSTASDMWSVGVILYILLSGCPPFHAA------TNQEKQQRILQGEFSFQEHTWKTITSSAKDLISSLLSVEPYKRPTASDLLMHPWVIGDCAKQDR-IDAEVVSKLQRFNARRKLRAAAIASVLSSKVALRTKRLR  350
MtCCaMK   ALSQGKITTKSDMWSLGVILYILLSGYPPFIAQ------NNRQKQQMIMNGNFSFYEKTWKGISQPAKNLISSLLTVDPSKRPSALELLSDPWVKGEKAKDVQ-MDPEIVSRLQSFNARRKLRAAAIASVWSSTIFLRTKKLK  354
```

```
OsCPK18   -------LNAEE-----LSDLRDQFNAIDVDKNGTISLEELKQALAKDVPWRLKGPRVLEIVEAIDSNTDGLVDFEEFVAATLHVHQLVEHDTEKWKSLSQAAFDKFDVDGDGYITSDELRM--QTGLKGS----------ID  470
TaCPK6    -------LNPEE-----LSDLRDQFNAIDIDKSGTISLEELKQALAKDVPWRLKGPRVLEIVEAIDSNTDGFVDFEEFVAATLHVHQLVEHDTEKWKSLSQAAFDKFDVDGDGYITSNELRM--NTGLKGS----------ID  469
MtCDPK1   -------LNEGE-----LSDLKDQFDAIDVDKNGAISLEEMRQALAKDLPWKLKESRVLEILQAIDSNTDGLVDFTEFVAATLHVHQLEEHDSDKWQQRSQAAFEKFDIDKDGYITPEELRM--HTGMRGS----------ID  517
OsCPK4    -------LKEEE-----LADLKDQFDAIDVDKSGSISIEEMRHALAKDLPWRLKGPRVLEIIQAIDSNTDGLVDFEEFVAATLHIHQMAELDSERWGLRCQAAFSKFDLDGDGYITPDELRMVQHTGLKGS----------IE  479
OsCPK30   -------LTANQ-----LFYLREQFELLGPNKNGYISLQNLKTALVKNSTDAMKDSRVIDFVNTVCTLQYRKLDFEEFAASAVSVYQMEALET--WEQHARRAYELFDKEGNRPIVIEELASELGLGPSVP----------LH  560
OsCPK3    -------LSAEE-----VEDIKEMFKAMDTDNDGIVSYEELKSGIAK-FGSHLAESEVQMLIEAVDTNGKDALDYGEFLAVSLHLQRMANDEH------LRRAFLFFDKDGNGYIEPEELREALVDDGAGD------SMEVVN  492
TaCPK3    -------LSAEE-----VEDIKEMFKAMDTDNDGIVSCEELKSGIAK-FGSHLAESEVQMLIEAVDTNGKGVLDYAEFLAVSLHLQRMANDEH------LRRAFLFFDKDGNGYIEPDELREALKDDGAAD------SMEVVN  486
OsCPK16   -------LSAEE-----VEDIKDMFKVMDTDNDGIVSYEELKSGIAK-FGSHLAESEVQMLIEAVDTNGRGALDYGEFLAVSLHLQRMTNGEH------LRRAFLFFDKDGNGYIEPEELQEALVEDGATD------IMEVVK  488
TaCPK15   -------LSAEE-----VEDIKEMFKVMDTDNDGIVSYEELKSGIAK-FGSHLAESEVQMLIEAVDTNGRGALDYGEFLAVSLHLQRMANDEH------LRRAFLFFDKDGDGFIEPEELQEALAEDGAVD------ITEVVK  492
OsCPK8    -------LSAEE-----AADIKDMFDKMDVSKNGQLTFEDFKAGIRK-LGNQMPDSDLKILMDAADIDKNGILDYQEFVAVSIHVRKIGNDEH------IQKAFSYFDQNKSGYIEIEELREALVDEIDGN------DEDIIN  479
TaCPK12   -------LSLEE-----VADIKKMFDSMDVNNKGQLTFEEFKAGLRK-LGNKMHDSDLQMLMDAADVDKNGTLDYGEFVAVSIHVRKIGNDEH------IQKAFSYFDQDKSGYIEIEELRVALTDEVDGAC-----DEDIIN  476
OsCPK20   -------LSVEE-----VAGIKDMFEKMDLNKDNMINFDELKLGLHK-LGHQMADADVQILMDAADVDGNGSLDYGEFVALSVHLRKIGNDEH------LHKAFAYFDRNQSGYIEIDELRESLADDLGAN------HEEVIN  490
TaCPK7    -------LSVEE-----VAGIKDMFEKMDLNKDSMINFDELKLGLNK-LGHQMPDADVQILMDAADADGNGCLDYGEFVTLSVHLKKIGNDEH------LHKAFGYFDRNKSGYIEIDELRESLADDLGPN------HEEVIN  483
OsCPK9    -------LSVEE-----VEVIKDMFALMDTDNNGRVTLQELKDGLTK-VGSKLAEPEMELLMEAADVDGNGYLDYGEFVAVTIHLQRLSNDNH------LRTAFLFFDKDGSGYIDRAELADALADDSGHA------DDAVLD  516
TaCPK19   -------LSAEE-----VEVIKEMFALMDTDNNGRVTLDELKAGLAR-VGSKLAEPEMELLMEAADVDGDGYLDYAEFVAITIHLQRLSNDQH------LRKAFLFFDRDSSGYIERPELADALADDSGKA------DDAVVD  511
OsCPK21   -------LPGEE-----VDKYVQMFHHMDKDKNGHLSLDELLEGLHI-NGQPVPEPEIRMLLEAADTDGNGTLDCDEFVTVSVHLKKMSNDEY------LAAAFNYFDKDGSGFIELDELR----EEVGP-------NEQAIL  510
OsCPK22   -------LPVEE-----MDKYTQMFHKMDKDNSGNLTLEDLKLGLQI-NGHPVPETEIEMLLEAGDIDGNGTLDCEEFVTVLLHIKKMSNEEY------LPKAFKFFDKDGNGFIEMEELMDALGDELGP-------TEQVVK  524
OsCPK29   -------LPTEE-----LDAIRELFNMLDTKKKGHLTLEELRKGLQV-IGHNIHDTDVDMLMEAADIDGNGILDCKEFVTVSIHLKKIRSDEH------LPKVFSFFDKNGSGYIEIEELK----EALSPRG-----DQKSID  496
OsCPK1    -------LSDEE-----ITGLKEMFRSLDTDNSGTITLEELRSGLPK-LGTKISESEIRQLMEAADVDGNGTIDYAEFISATMHMNRLEKEDH------ILKAFEYFDKDHSGYITVDELEEALKKYDMGD-------DKTIK  480
TaCPK8    -------LSEEE-----ITGLKEMFRSLDTDNSGTITLEELRSGLPK-LGTKISESEITQLMEAADVDGNGTIDYSEFVSATIHMNRLEKEDH------ILKAFEYFDKDHSGYITVDELEEALKKYDMGD-------DKTNK  480
OsCPK15   -------LSEEE-----IVGLKEMFKSLDTDNSGTITLEELRAGLPK-LGTKISESELRQLMEAADVDGNGSIDYVEFISATMHMNRLEKEDH------IYKAFEYFDKDHSGFITVDELEEALTKYDMGD-------EATIK  504
OsCPK2    -------LSEEE-----IRGLKEMFKSMDSDNSGTITVDELRKGLSK-QGTKLTEVEVQQLMEAADADGNGTIDYDEFITATMHMNRMDREEH------LYTAFQYFDKDNSGCISKEELEQALREKGLLD-------GRDIK  475
TaCPK13   -------LSEEE-----IRGLKEMFKSXXXDNSGTITVDELRKGLGK-QGTKLTEAEVEQLMEAADADGSGTIDYDEFITATMHMNRMDREEH------LYTAFQYFDKDNSGYISKEELEQALREKGLLA-------GRDMS  477
OsCPK14   -------LSEEE-----IRGLKEMFKSMDSDNSGTITVDELRKGLAK-KGTKLTEAEVQQLMEAADADGNGTIDYEEFITATMHMNRMDREEH------LYTAFQYFDKDNSGYITIEELEQALREKGLMD-------GREIK  488
OsCPK25   -------LSEEE-----IKGLKEMFKNIDKDNSGTITLEELKNGLAK-QGTKFSDNEIEQLMEAADADGNGIIDYEEFVTATVHMNKMDREEH------LYTAFQYFDKDNSGYITKEELEQALKEQGLYD-------ANEIK  497
OsCPK26   -------LSEEE-----IKGLKEMFKNIDKDNSGTITLEELKNGLAK-QGTKFSDNEIEQLMEAADADGNGIIDYEEFVTATVHMNKMDREEH------LYTAFQYFDKDNSGYITKEELEQALKEQGLYD-------ANEIK  497
OsCPK12   -------LSPEE-----IKGLKQMFNNMDTDRSGTITVEELKVGLTK-LGSRISEAEVQKLMEAVDVDKSGSIDYSEFLTAMINKHKLEKEED------LLRAFQHFDKDNSGYITRDELEQAMAEYGMGD-------EANIK  504
TaCPK18   -------LSPEE-----IKGLKQMFNNMDTDKSGTITVEELKIGLTK-LGSKITEAEVQKLMEAVDVDKSGSIDYTEFLTAMMNKHKLEKEED------LLHAFQHFDKDNSGYISREELEQAMTEYGMGD-------EANIK  507
OsCPK19   -------LNEEE-----IKGLKQMFTNMDTDNSGTITYEELKAGLAK-LGSKLSEAEVKQLMEAADVDGNGSIDYVEFITATMHRHKLERDEH------LFKAFQYFDKDNSGFITRDELESALIEHEMGD-------TSTIK  498
TaCPK5    -------LNEEE-----IKGLKQMFMNMDTDNSGTITYEELKAGLAK-LGSKLSEAEVKQLMDAADVDGNGSIDYVEFITATMHRHKLERDEH------LFKAFQYFDKDNSGFITRDELETALIEHEMGD-------ADTIK  498
ZmCDPK2   -------LNEEE-----IKGLKQMFMNMDTDNSGTITYEELKAGLAK-LGSKLSEAEVKQLMEAADVDGNGSIDYVEFITATMHRHKLERDEH------LFKAFQYFDKDNSGFITRDELESALIEHEMGD-------TSTIR  479
ZmCDPK9   -------LNEEE-----IKGLKQMFMNMDTDNSGTITYEELKAGLAK-LGSKLSEAEVKQLMEAADVDGNGSIDYVEFITATMHRHKLERDEY------LFKAFQYFDKDNSGFITRDELESALIEHEMGD-------TSTIK  497
MtCDPK3   -------MSEEE-----IRGLKAMFTNMDTDNSGTITYEELKAGLQR-LGSKLSEAEVKQLMEAADVDGNGTIDCIEFITATMHRHKLERDDH------LYKAFQYFDKDSSGFITRDELETAMKEYGMGD-------DATIK  497
MsCDPK3   -------MSEEE-----IRGLKAMFTNMDTDNSGTITYEELKAGLQR-LGSKLSEAEVKQLMEAADVDGNGTIDCIEFITATMHRHKLERDDH------LYKAFQYFDKDSSGFITRDELETAMKEYGMGD-------DATIK  501
OsCPK10   -------LSEDE-----IAGLKEMFKMIDTDNSGQITFEELKVGLKK-VGANLQESEIYALMQAADVDNSGTIDYGEFIAATLHMNKIEREDH------LFAAFQYFDKDGSGYITADELQLACEEFGLGD--------VQLE  546
ZmCPK10   -------LSEDE-----IAGLREMFKMIDADNSGQITFEELKVGLEK-VGANLQESEIYALMQAADVDNNGTIDYGEFIAATLHLNKVEREDH------LFAAFQYFDKDGSGYITADELQVACEEFGLGD--------VQLE  566
OsCPK17   -------LSEDE-----IARLREMFKMLDTDNSGQITLEELKTGLRR-VGANLKDSEITTLMEAADIDNSGSIDYGEFIAATMHLNKVEREDN------LFAAFSYFDKDSSGYITQDELQKACEEFGIGD--------AHLE  519
TaCPK9    -------LSEDE-----IAGLREMFKMLDTDNSGQITLEELKTGLQR-VGANLKESEIATLMEAADIDNSGSIDYGEFLAATLHLNKVEREDN------LFAAFSYFDKDGSGYITQDELQKACEEFGIGD--------AHLD  513
OsCPK11   -------LSEEE-----IAGLKEMFKMLDTDNSGHITLEELKTGLQR-VGANLMDSEIDALMEAADIDNSGTIDYGEFIAATLHINKVEKEDK------LFAAFSYFDKDGSGYITQDELQKACEEFGIGD--------TRIE  526
OsCPK27   -------LSEEE-----IAGLKEMFKMMDTDNSGQINYEELKAGLER-VGANMKESEIYQLMQAADIDNSGTIDYGEFIAATLHLNKVEREDH------LYAAFQYFDKDGSGYITSDELQQACDEFGIED--------VRLE  563
OsCPK24   -------LSEEE-----IGGLKELFKMIDTDDSGTITFDELKEGLKR-VGSELTEHEIQALMEAADIDNSGTIDYGEFIAATLHMNKLEREEN------LVSAFSFFDKDGSGFITIDELSQACREFGLDD--------LHLE  460
TaCPK4    -------LSEEE-----IGGLRELFKMIDADNSGTITFDELKDGLKR-VGSELTEHEIQALMDAADIDNSGTIDYGVFIAATLHMNKLEREEN------LVSAFSFFDKDGSGFITIDELSQACRQFGLDD--------VHLE  461
ZmCPK11   -------LSEEE-----IGGLKELFKMIDTDSSGTITFDELKDGLKR-VGSELTENEIQALMEAADIDNSGTIDYGEFIAATLHMNKLEREEN------LVSAFSFFDKDGSGFITIDELSQACREFGLDD--------LHLE  457
OsCPK28   -------LSEEE-----IGGLKELFKMIDTDNSGTITYDELKNGLKR-VGSDLMEPEIQALMDAADIDNSGTIDYGEFLAATLHMNKLEREEN------LVSAFTFFDKDGSGFITIDELSQACEQFGLSD--------VHLE  462
OsCPK13   -------LSEEE-----IAGLKEMFQTMDADNSGAITYDELKEGLRK-YGSTLKDTEIRDLMDAADIDNSGTIDYIEFIAATLHLNKLEREEH------LVAAFSYFDKDGSGYITVDELQQACKEHNMPD--------AFLD  501
TaCPK2    -------LSEEG-----IAGLKEMFQTMDADNSGAITYDELKEGLRK-YGSTLKDTEIRDLMDAADVDNSGTIDYIEFIAATLHLNKLEREEH------LVAAFSYFDKDGSGYITVDELQQACQEHNMPD--------AFLD  508
ZmCDPK1   -------LSEEE-----IAGLKEMFQTMDTDNSGAITYDELKEGLRK-YGSTLKDTEIRDLMDAADIDNSGTIDYIEFIAATLHLNKLEREEH------LVAAFSYFDKDGSGYITVDELQLACKEHNMPD--------AFLD  440
ZmCDPK7   -------LSEEE-----IAGLKEMFQTMDTDNSGAITYDELKEGLRK-YGSTLKDTEIRDLMDAADIDNNGTIDYIEFIAATLHLNKLEREEH------LVAAFSYFDKDGSGYITVDELQQACKEHNMPD--------AFLD  504
OsCPK5    -------LSEEE-----IAGLKEMFKAMDTDNSGAITYDELKEGMRK-YGSTLKDTEIRDLMEAADVDNSGTIDYIEFIAATLHLNKLEREEH------LVAAFSYFDKDGSGYITVDELQQACKEHNMPD--------AFLD  505
OsCPK7    -------LSEEE-----IAGLREMFKAVDTKNRGVITFGELREGLRR-FGAEFKDTEIGDIMEAAHNDNNVTIHYEEFIAATLPLNKIEREEH------LLAAFTYFDKDGSGYITVDKLQRACGEHNMED--------SLLE  492
TaCPK1    -------LSEEE-----IAGLRELFKTVDIKNRGVITFGELRKGLTR-YGNELVDTEICDIMEAADTDTDVTINYEEFIAATMPLNKIEREEH------LKAAFTYFDKDGSGYITVDKLQRACAEYNMEG--------TLLE  494
OsCPK23   -------LSEEE-----IVGLREMFKAMDTKNRSVVTFGEL-KGLKR-YSSVFKDTEINDLMEAAD-DTTSTINWEEFIAAAVSLNKIEREKH------LMAAFTYFDKDGSGFITVDKLQKACMERNMED--------TFLE  484
OsCPK6    -------LSEEE-----LAGLKEMFKAMDTDASGAITFDELKEGLRR-YGSNLREAEIRDLMDAADVDKAAPS------TTTSSSPPPSTSTS------WSARSTCSPPSPTSTATAAATSPSTSSS----------------  473
OsCPK31   -------MRQRGKAPSFNSALQELFAAYDKDKSGTISFEELAEGLRG-QGYVVNESEVRQLMEKMDMDHDGNVGGDEFLATLIDWGQVMQEQE--WQSYVDQAFNRMDLDGDGFIDLDELLSELPAAYFHE-------PSSED  529
OsCCaMK   NLLGTHDLTSEE-----LDNLRLHFGRICADGEN-ATLSEFEQVLRA-MKMDSLIPLAPRVFDLFDNNRDGTVDMREILCGFSSLRNSRGDDA------LRLCFQMYDADRSGCISKEELASMLRALPEECLPGDITEPGKLD  476
TaCCaMK   SLLGTHDLSSEE-----LDNLRQHFARICADGEN-ATLTEFEQVLKA-MKMDSLIPLAPRVFDLFDNNRDGTVDMREILCGLSSLRNSRGDNA------LRLCFQMYDADRSGCISKEELASMLRALPEECLPGDIAEPGKLD  480
MtCCaMK   SLVGSYDLKEEE-----IENLRMHFKKICADRDN-ATLSEFEEVLKA-MNMLSLIPFASRIFDLFDNNRDGTVDMREILCGFSSLKNSKGEDA------LRLCFQMYDTDRSGCISKEEVASMLRALPYDCLPTDITEPGKLD  484
```

```
OsCPK18  PLLEEAD-------IDRDGKISLDEFRRLLKTA---------------------SMSSRNVQTPRSVHRS-------------------  512
TaCPK6   PLLEEAD-------IDKDGKISLDEFRRLLKTA---------------------SMSSRN-PTPRSVSK-------------------  509
MtCDPK1  PLLEEAD-------IDKDGKISLPEFRRLLRTA---------------------SIGSRNVTSPTLRHRRI-----------------  560
OsCPK4   PLLEEAD-------IDKDGRISLSEFRKLLRTA---------------------SMSNLPSPRGPPNPQPL-----------------  522
OsCPK30  VVLQDWI-------RHADGKLSFLGFIKLLH-----------------------GVSSRSIPKA------------------------  594
OsCPK3   DILQEVD-------TDKDGKISYDEFVAMMKTGTDWRKASRHYSR-----GRFNSLSMKLIKDGSVKLVNE-----------------  551
TaCPK3   DILQEVD-------TDKDGKISYDEFVAMMKTGTDWRKASRHYSR-----GRFNSLSMKLVKDGSVKLGVE-----------------  545
OsCPK16  DILQEVD-------TDKDGKISYEEFVAMMKTGTDWRKASRHYSR-----GRFNSLSIRLIKDGSVKLGNE-----------------  547
TaCPK15  DILQEVD-------TDKDGKISFEEFVAMMKTGTDWRKASRHYSR-----GRFNSLSIRLIKDGSVKMGNE-----------------  551
OsCPK8   SIIRDVD-------TDKDGKISYDEFAVMMKAGTDWRKASRQYSR-----QRFSNLSLKLQKDGSISDDTQ-----------------  538
TaCPK12  GIIHDVD-------TDKDGKISYDEFAAMMKAGTBWRKASRQYSR-----QRFSNLSLKLHKDGSISDDQK-----------------  535
OsCPK20  AIIRDVD-------TDKDGKISYDEFAAMMKAGTDWRKASRQYSR-----ERFTSLSLKLQKDGSLQLTTTQ----------------  550
TaCPK7   AIIRDVD-------TDKDGKISFDEFVAMMKAGTDWRKASRQYSR-----ERFTSLSLKLQKDGSLQITSTQ----------------  543
OsCPK9   HILREVD-------TDKDGRISYEEFVAMMKSGTDWRKASRQYSR-----ERFKTLSNSLIKDGSITMAR------------------  574
TaCPK19  HVLLEVD-------TDKDGRVSFEEFVAMMKAGTDWRKASRQYSK-----QRFKSLSNSLIKDGSISMAP------------------  569
OsCPK21  EILRDVD-------TDKDGRISYQEFELMMKSGADWRNASRHFSR-----ANFSTLSRRLCKDTLTP---------------------  565
OsCPK22  DIIRDID-------TDKDGRISYQEFESMMISGSDWRNASRRYSK-----ANFSSLSRKLCKGNS-----------------------  577
OsCPK29  DIFLDVD-------IDKDGKISYEEFELMMSAGMDWRNASRQYSR-----AVYNTLSRKIFKEVSLKLDHSGPLVAAGK----------  563
OsCPK1   EIIAEVD-------TDHDGRINYQEFVAMMRNN-----------------NPE-IAPNR-RRMF-------------------------  518
TaCPK8   DIIAEVD-------TDHDGRINYQEFVAMMRNN-----------------SPE-IVPNR-RRMF-------------------------  518
OsCPK15  EIIAEVD-------TDHDGRINYQEFVAMMKNN-----------------SPE-IVPNR-RRMF-------------------------  542
OsCPK2   DIISEVD-------ADNDGRIDYSEFAAMMRKG-----------------NPE-ANPKK-RRDVVI-----------------------  515
TaCPK13  EIVSEVD-------ADNDGRIDYSEFVAMMRKG-----------------APEGANPKK-RRDVVL-----------------------  518
OsCPK14  DIISEVD-------ADNDGRINYTEFVAMMRKG-----------------DPE-ANPKK-RRDVVL-----------------------  528
OsCPK25  DVITDAD-------SNNDGRIDYSEFVAMMRKGS----------------GCAEATNPKKKRRDLVL----------------------  541
OsCPK26  DVITDAD-------SNNDGRIDYSEFVAMMRKGS----------------GCAEATNPKKKRRDLVL----------------------  541
OsCPK12  QVLDEVD-------KDKDGRIDYEEFVEMMRKG----------------------IQT--------------------------------  533
TaCPK18  AVLDEVD-------KDRDGNIDYEEFVEMMRKG----------------------K---------------------------------  534
OsCPK19  DIISEVD-------TDNDGRINYEEFCAMMRGGG---------------------MQQPMRLK--------------------------  533
TaCPK5   DIISEVD-------TDNDGRINYEEFCAMMRGG----------------------MQQPIRLK--------------------------  532
ZmCDPK2  EIISEVD-------TDNDGRINYEEFCAMMRGG----------------------MQQPMRLK--------------------------  513
ZmCDPK9  EIISEVD-------TDNDGRINYEEFCAMMRGG----------------------MQQPMRLK--------------------------  531
MtCDPK3  EIISEVDTIISEVDTDHDGRINYEEFCAMMRSG----------------------NQQQVKLF--------------------------  538
MsCDPK3  EIISEVDTIISEVDTDHDGRINYEEFCAMMRSG----------------------NQQQVKLF--------------------------  542
OsCPK10  EMIREVD-------EDNDGRIDYNEFVAMMQ----KPTMGLPAKKSGGLQNSFS-IGFREALRMSWETATRVSHTLVEATTTRSICCLP  623
ZmCPK10  DLIGEVD-------QDNDGRIDYNEFVAMMQ----KPTVGGSRRRPICRTASASGSASGSGRRSGWPRPLCLWLPCCLRVGVDD-----  639
OsCPK17  DIIKDID-------QDNDGRIDYNEFVTMMR----KGNNPLGKKG----QGQLS-FGLREALKLG------------------------  568
TaCPK9   DIIRDID-------QDNDGRIDYNEFVTMMQ----KGNNPLGKKG----QGQMS-FGLREALKIR------------------------  562
OsCPK11  DIIGDID-------QDNDGRIDYNEFVEMMQ----KGNNAMGKMG----QHSTGNFGLGEALKLR------------------------  576
OsCPK27  DMIGEVD-------QDNDGRIDYNEFVAMMQ----KTTTGFGKKG----GHNFS--GFRDALKSHS-----------------------  612
OsCPK24  DMIKDVD-------QNNDGQIDYSEFTAMMR----KGNAG-----------GAGRRTMRNSLQLNLGEILNPSNS--------------  513
TaCPK4   DMIKDVD-------QNNDGQIDYSEFTAMMR----KGNAG-----------ATGRRTMRNSLNLNLGDILNPSNS--------------  514
ZmCPK11  DMIKDVD-------QNNDGQIDYSEFTAMMR----KGNAG-----------ATGRRTMRNSLHLNLGELLNPSKT--------------  510
OsCPK28  DMIKDVD-------QNNDGQIDYSEFAAMMR----KGNAGGANAGGVTSTGGTGRRTMRNSLRVNLGDILKPNEN--------------  526
OsCPK13  DVINEAD-------QDNDGRIDYGEFVAMMT----KGNMGVGRRT----MRNSLNISMRDAPGAL------------------------  551
TaCPK2   DVIKEAD-------QDNDGRIDYGEFVAMMT----KGNMGVGRRT----MRNSLNISMRDAPGAI------------------------  558
ZmCDPK1  DVINEAD-------QDNDGRIDYGEFVAMMT----KGNMGVGRRT----MRNSLNISMRDDLVCSET----------------------  492
ZmCDPK7  DVINEAD-------QDNDGRIDYGEFVAMMT----KGNMGVGRRT----MRNSLNISMRDAPGAF------------------------  554
OsCPK5   DVIKEAD-------QDNDGRIDYGEFVAMMT----KGNMGVGRRT----MRNSLNISMR------------------------------  549
OsCPK7   EIISEVD-------QNNDGQIDYAEFVAMMQ----GSNVGLGWRT----MESSLNVALRDAPQVH------------------------  542
TaCPK1   EIILEAD-------QNNDGQIDYAEFVAMMQGNTNGGNIGLGRPT----METSLNVTLRDAAQVH------------------------  548
OsCPK23  EMILEVD-------QNNDGQIDYAEFVTMMQ----SNNFGLGWQT----VESSLNVALREAPQVY------------------------  534
OsCPK6   ---------------TPAGTTTWPTLASTTS---------SAKST----KTTTAASTTASSSQ--------------------------  508
OsCPK31  ERISEAKRMLREADENGDGRISKQEFYNLLRDN-----------------VAPDSLSMYDDRLSHNVAAMSV-----------------  584
OsCCaMK  EVFDQMD-------ADSDGKVTFDEFKAAMNKD----------------------SALQDVLLSSLRPQ--------------------  516
TaCCaMK  EMFDTMD-------ANGDGEVTFDEFKAAMQKG----------------------SALQDEVLSSLRPGQQ------------------  522
MtCCaMK  EIFDLMD-------ANNDGKVTFDEFKAAMQRD----------------------SSLQDVVLSSIRP---------------------  523
```
