# Supplementary material for: A rice calcium-dependent protein kinase is expressed in cortical root cells during the presymbiotic phase of the arbuscular mycorrhizal symbiosis
Source: BMC Plant Biol. 2011 May 19;11:90. doi: 10.1186/1471-2229-11-90 (PMC3125349; doi:10.1186/1471-2229-11-90)
Supplement: Additional file 5 — Table S1 and Table S2. Table S1. Symbiosis-related cis-elements identified in the OsCPK18, OsCPK4, MtCPK1, OsCCAMK and MtCCaMK promoters. The 2 kb upstream region of each promoter was analyzed by using the PLACE motif database. Table S2. Number of symbiosis-related cis-elements present in the OsCPK18, OsCPK4, MtCPK1, OsCCaMK and MtCCaMK promoters. The presence (x) or absence (-) of the indicated motifs within the 2 kb upstream region of each promoter is shown. Multiple copies of a given cis-element were present in a particular promoter (2x, 3x, 4x, etc.). The PLACE motif database was used to perform this analysis. Motifs are listed by alphabetical order. [file 1471-2229-11-90-S5.PDF]

Table S1. Symbiosis-related *cis*-elements identified in the *OsCPK18, OsCPK4, MtCPK1, OsCCaMK* and *MtCCaMK* promoters

cis-Element	Sequence	Function feature
**ABRE-related consensus motif**	(C/A)ACG(T/C)G(T/G/C)	"ABRE-related sequence" or "Repeated sequence motifs" Identified in the upstream regions of 162 Ca(2+)-responsive up-regulated genes.
ASF1 MOTIF	TGACG	TGACG motifs are found in many promoters and are involved in transcriptional activation of several genes by biotic and abiotic stress.
**CGCG-BOX**	(A/C/G)CGCG(G/T/C)	"CGCG box" found in promoters of many genes under Ca++/calmodulin regulation.
GCC-CORE	GCCGCC	Core of GCC-box found in many pathogen-responsive genes such as PDF1.2, Thi2.1, and PR4.
GT1-CONSENSUS	G(A/G)(A/T)AA(A/T)	Binding of GT-1-like factors to the PR-1a promoter influences the level of SA-inducible gene expression
GT1-GMSCAM4	GAAAAA	"GT-1 motif" found in the promoter of soybean CaM isoform; Plays a role in pathogen- and salt-induced SCaM-4 gene expression
**NODCON1GM**	AAAGAT	One of two putative nodulin consensus sequences
**NODCON2GM**	CTCTT	One of two putative nodulin consensus sequences
**OSE1ROOTNODULE**	AAAGAT	One of the consensus sequence motifs of organ-specific elements (OSE) characteristic of the promoters activated in infected cells of root nodules and in the arbuscule-containing cells of mycorrhizal roots
**OSE2ROOTNODULE**	CTCTT	One of the consensus sequence motifs of organ-specific elements (OSE) characteristic of the promoters activated in infected cells of root nodules and in the arbuscule-containing cells of mycorrhizal roots
**P1BS**	G(G/A/C/T)ATAT(G/A/C/T)C	PHR1-binding sequence found in the upstream regions of phosphate starvation responsive genes from several plant species
SEBFCONSST PR10A	(T/C)TGTC(A/T)C	Binding site of the potato silencing element binding factor (SEBF) gene found in promoter of pathogenesis-related gene PR-10a.
WB BOX PC WRKY1	TTTGAC(T/C)	"WB box"; WRKY proteins bind specifically to the W box (T)(T)TGAC(C/T). Found in PR1 gene in parsley
W BOX ATNPR1	TTGAC	"W-box" found in promoter of Arabidopsis thaliana NPR1 gene.
WRKY71 OS	TGAC	"A core of TGAC-containing W-box". Binding site of rice WRKY71.

Table S2. Number of *cis*-elements in the 2kb upstream region of the rice *cpk* and *SYM* genes

cis-Element	Oscpk18	Oscpk4	MtCPK1	OsCCamK	MtCCaMK
**ABRE-related**	4x	1x	-	-	3x
ASF1 MOTIF	4x	1x	-	3x	-
**CGCG- BOX**	6x	-	-	-	6x
GCC-CORE	1x	-	-	-	-
GT1-CONSENSUS	17x	21x	23x	13x	20x
GT1-GMSCAM4	3x	8x	6x	3x	8x
**NODCON1GM**	-	-	3x	3x	3x
**NODCON2GM**	5x	6x	5x	7x	9x
**OSE1ROOTNODULE**	-	-	3x	3x	3x
**OSE2ROOTNODULE**	5x	6x	5x	7x	9x
**P1BS**	-	2x	6x	-	4x
SEBFCONSST PR10A	1x	1x	2x	2x	-
WB BOX PC WRKY1	2x	-	3x	2x	-
W BOX ATNPR1	4x	7x	6x	5x	2x
WRKY71 OS	15x	11x	12x	17x	6x
