# Supplementary material for: A rice calcium-dependent protein kinase is expressed in cortical root cells during the presymbiotic phase of the arbuscular mycorrhizal symbiosis
Source: BMC Plant Biol. 2011 May 19;11:90. doi: 10.1186/1471-2229-11-90 (PMC3125349; doi:10.1186/1471-2229-11-90)
Supplement: Additional file 6 — Table S3: Primer sequences used in the real-time qPCR analysis. [file 1471-2229-11-90-S6.PDF]

Table S3. Primers used for quantitative reverse transcriptase-polymerase chain reaction (RT-qPCR) analysis of *cpk* genes.

Gene	Accesion Number	Primer sequence
OsCPK4	AK060738	**Forward:** 5′-CGTGTGCAGCATGCAGATAA -3′
		**Reverse:** 5′- TGCGATGAATACGTGCAATCA-3′
OsCPK18	AK121471	**Forward:** 5′- CACAAAAGTGAACAAAGGGCAGT -3′
		**Reverse:**5′- CGTGTCGATGAAAAATATTACAACAAT -3′
OsCCaMK	AK070533	**Forward:** 5′- GCAAGGTCACCTTCGACGAG -3′
		**Reverse:** 5′- GGGCGCTGTCCTTGTTCAT -3′
OsAct1	AK100267	**Forward:** 5′- GGCATCTCTCAGCACATTCCA -3′
		**Reverse:** 5′- TCGTACTCAGCCTTGGCAATC -3′
