# Supplementary material for: A rice calcium-dependent protein kinase is expressed in cortical root cells during the presymbiotic phase of the arbuscular mycorrhizal symbiosis
Source: BMC Plant Biol. 2011 May 19;11:90. doi: 10.1186/1471-2229-11-90 (PMC3125349; doi:10.1186/1471-2229-11-90)
Supplement: Additional file 7 — Information of the RT-qPCR analysis based on the MIQE (Minimum Information for Publication of Quantitative Real-Time PCR Experiments) checklist [55]. [file 1471-2229-11-90-S7.PDF]

The information of the RT-qPCR analysis based on the MIQE checklist

**EXPERIMENTAL DESIGN**
Definition of experimental and control groups
Number within each group
Assay carried out by core lab or investigator's lab?
Acknowledgement of authors' contributions
**SAMPLE**
Description
Volume/mass of sample processed
Microdissection or macrodissection
Processing procedure
If frozen - how and how quickly?
If fixed - with what, how quickly?
Sample storage conditions and duration (especially for FFPE samples)

Experimental design is provided in the material and method section. Total RNA was extracted from liquid nitrogen frozen whole roots at different times after inoculation with *G. intraradices* spores, as well as from mock-inoculated roots. For each time point, roots from at least 12 individual plants were collected. Three independent experiments were carried out. About 0.5-1g of samples was used for RNA extraction.

**NUCLEIC ACID EXTRACTION**
Procedure and/or instrumentation
Name of kit and details of any modifications
Source of additional reagents used
Details of DNase or RNAse treatment
Contamination assessment (DNA or RNA)
Nucleic acid quantification
Instrument and method
Purity (A260/A280)
Yield
RNA integrity method/instrument
RIN/RQI or Cq of 3' and 5' transcripts
Electrophoresis traces
Inhibition testing (Cq dilutions, spike or other)

The RNA isolation procedure was done using the TRIZOL®Reagent (Invitrogen, Carlsbad, CA, USA). The extraction was performed following manufacturer’s instructions. To remove any remaining DNA traces, a DNase-treatment was performed. 10µg RNA was treated with 10 units of RNAse-free DNase (Roche, Mannhein, Germany) in a 100 µl final volume according to

manufacturer’s instructions. After 5 minutes heat inactivation of the enzyme and precipitation with 0.1 vol. of 3M Na-acetate and 2.5 vol. cold ethanol (100%), the RNA was resuspended in 40 µl of sterile water and quantified using the NanoDrop 1000 spectrophotometer (NanoDrop, Wilmington, USA). The A260/280 ratio is generally between 1.9 and 2.0. RNA integrity was check by electrophoresis gel.
Contamination was assessed by several no RT control of different samples, using them in the qPCR reaction. Additionally, genes used for qPCR are flanking an intron and since a melting curve is performed as standard, a contamination would be visible as additional peak.

**REVERSE TRANSCRIPTION**
Complete reaction conditions
Amount of RNA and reaction volume
Priming oligonucleotide (if using GSP) and concentration
Reverse transcriptase and concentration
Temperature and time
Manufacturer of reagents and catalogue numbers
Cqs with and without RT
Storage conditions of cDNA

The first cDNA was synthesized from DNase-treated total RNA (1 µg) with M-MLV (Moloney-Murine Leukemia Virus) Reverse Transcriptase (Invitrogen, Carlsbad, CA, USA). 9.5 µl of RNA, 0.5 µl of RNAse inhibitor (Roche, Mannhein, Germany) and 1 µl of oligo$(dT)_{18}$ (500 µg/ml) were incubated at 70°C for 10 min, then 5 min at room temperature and then chilled on ice. All other steps were performed according to manufacturer’s instructions. The incubation time at 37°C was increased from 50 to 60 min. Aliquots of the resulting RT reaction product were used as template for PCR analysis. In no RT control samples (RNA samples without RT enzyme) no amplification was detected. cDNA was stored in eppendorf tubes at -20°C.

**qPCR TARGET INFORMATION**
If multiplex, efficiency and LOD of each assay.
Sequence accession number
Location of amplicon
Amplicon length
In silico specificity screen (BLAST, etc)<br>Pseudogenes, retropseudogenes or other homologs?<br>Sequence alignment
Secondary structure analysis of amplicon
Location of each primer by exon or intron (if applicable)
What splice variants are targeted?

Multiplex qPCR was not performed. Sequence accession numbers are included in table S2 in Additional File 1. Gene-specific primers for selected *CPK* genes were designed using the Primer Express software (Applied Biosystems, Norwalk, CT, USA). Primer sets spanning intron regions

for each *CPK* gene were designed to confirm that the RT-PCR products were from RNA transcripts rather than from genomic DNA. All primers (listed in Table S2 in Additional File 1) were designed to amplify 70-100 bp of the N-terminal region of the genes. *In silico* screen were performed with NCBI Blast and can be obtained from above web side.

**qPCR OLIGONUCLEOTIDES**
Primer sequences
RTPrimerDB Identification Number
Probe sequences
Location and identity of any modifications
Manufacturer of oligonucleotides
Purification method

Primer sequences are included in the manuscript as Table S2 in Additional File 1. No modifications were used. Primers were purchase from Integrated DNA Technologies, Inc. (IDT), Coralville, Iowa.

**qPCR PROTOCOL**
Complete reaction conditions
Reaction volume and amount of cDNA/DNA
Primer, (probe), Mg++ and dNTP concentrations
Polymerase identity and concentration
Buffer/kit identity and manufacturer
Exact chemical constitution of the buffer
Additives (SYBR Green I, DMSO, etc.)
Manufacturer of plates/tubes and catalog number
Complete thermocycling parameters
Reaction setup (manual/robotic)
Manufacturer of qPCR instrument

Quantitative real time PCR (RT-qPCR) analyses were carried out in optical 96-well plates in a LightCycler® 480 Real-Time PCR System (Roche) according to the following program:
10 min at 95 °C,
followed by 45 cycles of
95 °C for 10 s,
60 °C for 30 s,
and an additional cycle of dissociation curves to ensure an unique amplification.

The reaction mixture (in a final volume of 20 µl) contained:
2 µl cDNA sample,
10 µl 2X SYBR Green Master mix reagent (Roche, Mannhein, Germany),
300 µM of each gene-specific primers.
Reactions were set up manually in a designated room using designated equipment.

**qPCR VALIDATION**
Evidence of optimisation (from gradients)
Specificity (gel, sequence, melt, or digest)
For SYBR Green I, Cq of the NTC
Standard curves with slope and y-intercept
PCR efficiency calculated from slope
Confidence interval for PCR efficiency or standard error
r2 of standard curve
Linear dynamic range
Cq variation at lower limit
Confidence intervals throughout range
Evidence for limit of detection
If multiplex, efficiency and LOD of each assay.

The specificity of the amplification products have been confirmed by size estimations on a 2% agarose gel, sequencing of the products and by analyzing their melting curves. Without a template, no Cq could be determined since it never passed the threshold line. Serial 4-fold dilution of cDNAs were used to calculate the standard curve and measure the amplification efficiency for each target and reference gene with the LightCycler® 480 SW 1.5 Software.

	**slope**	**y-intercept**	**efficiency**	**error**
OsCPK4	-3.625	21.52	1.887	0.0108
OsCPK18	-3.700	22.12	1.863	0.0150
OsCCaMK	-3.818	25.16	1.828	0.0195
OsAct1	-3.692	25.99	1.866	0.0127

**DATA ANALYSIS**
qPCR analysis program (source, version)
Cq method determination
Outlier identification and disposition
Results of NTCs
Justification of number and choice of reference genes
Description of normalisation method
Number and concordance of biological replicates
Number and stage (RT or qPCR) of technical replicates
Repeatability (intra-assay variation)
Reproducibility (inter-assay variation, %CV)
Power analysis
Statistical methods for result significance
Software (source, version)
Cq or raw data submission using RDML

qPCR analysis program (source, version): LightCycler® 480 Software, version 1.5.0.39;
Obtained data were analyzed using the comparative Ct (threshold cycle) method.

Cq's were determined by setting the threshold automatic

No data have been exclude from the calculations

Results of NTCs: no amplification products present thus no Cqs

Justification of number and choice of reference genes: cDNAs had previously been tested with another reference gene (*OsUbi1*) with the same results.

Description of normalization method: endogenous reference gene. Data were normalized with OsAct1 as internal control (The average CT values from triplicate PCRs were normalized to the average CT values for the OsAct1 gene from the same RNA preparations)

Number and concordance of biological replicates: Three independent biological replicates were analysed.

Number and stage (RT or qPCR) of technical replicates: 3 at qPCR level (routinely, three replicate reactions were used for each sample) , 2 for RT analysis.
