# Supplementary material for: A rice calcium-dependent protein kinase is expressed in cortical root cells during the presymbiotic phase of the arbuscular mycorrhizal symbiosis
Source: BMC Plant Biol. 2011 May 19;11:90. doi: 10.1186/1471-2229-11-90 (PMC3125349; doi:10.1186/1471-2229-11-90)
Supplement: Additional file 8 — Table S4: Primer sequences used in the RT-PCR analysis. [file 1471-2229-11-90-S8.PDF]

Table S4. Primers used for semi-quantitative reverse transcriptase-polymerase chain reaction RT-PCR analysis of rice *cpk* genes.

Gene	Accesion Number	Primer sequence
OsCPK2	AK112112	**Forward:** 5′- ACGGAACCATCGATTACGACGAGT -3′
		**Reverse:** 5′- TTCCATCGTTATCGGCGTCGACTT -3′
OsCPK4	AK060738	**Forward:** 5′- TGGCTGAGCTTGACTCTGAAAGGT -3′
		**Reverse:** 5′- TCTTGGACTGGGAAGGTTGCTCAT -3′
OsCPK7	AK066500	**Forward:** 5′-GGAAGCGGCACACAACGACAATAA -3′
		**Reverse:** 5′- TGCATCATGGCTACAAATTCGGCG -3′
OsCPK8	AK066615	**Forward:** 5′- GTTGCTGTGTCTATCCATGTGAGG -3′
		**Reverse:** 5′- TGACGGCAAACTCGTCGTAGCTTA -3′
OsCPK9	AK105102	**Forward:** 5′- GGCGTCGCGGCAATACT-3′
		**Reverse:** 5′-CATTGCATCGACCTCATATTCTCA -3′
OsCPK10	AK072204	**Forward:** 5′- CGAAATCGCCGGCCTGAAAGAAAT -3′
		**Reverse:** 5′- TTGTCGTCGCCTCTACAAGTGTGT -3′
OsCPK13	AK061881	**Forward:** 5′-ACATGCCCGATGCTTTTCT -3′
		**Reverse:** 5′-AAAATCGCAGGAGTTCGTTG -3′
OsCPK15	AK070346	**Forward:**5′-CAGTCGATGAATTGGAAGAAGCTCTGAC-3′
		**Reverse:** 5′- AAACATCCGCCGTCGATTTGGAAC -3′
OsCPK16	AK101942	**Forward:** 5′- AAAGGATGACAAATGGCGAGCACC -3′
		**Reverse:** 5′- TAATGCCGTGACGCCTTTCTCCAA -3′
OsCPK17	AK068414	**Forward:** 5′- AAAGCCTGTGAGGAATTTGG -3′
		**Reverse:** 5′- TCCATTCTACATTCAGGGGTAGTT -3′
OsCPK18	AK121471	**Forward:** 5′- TGTGCATCAGCTAGTGGAACATGA -3′
		**Reverse:** 5′- CCTCGGAGTTTGTACATTGCGTGA -3′
OsCPK19	AK074028	**Forward:** 5′- CTGCCACAATGCATAGACACAAGC -3′
		**Reverse:** 5′- CCCTCCTCTCATCATAGCGCAAA -3′
OsCPK22	AK100474	**Forward:** 5′- CCAAGAATTTACCGGTGGAGGAGA -3′
		**Reverse:** 5′- ACCGTTACAAACTCCTCGCAATCT -3′
OsCPK24	AK102308	**Forward:** 5′- TTGATGAGCTGTCACAAGCA -3′
		**Reverse:** 5′- GGGGTTCAAGATCTCACCAA -3′
OsCPK25	Os11g04170	**Forward:** 5′- TGCTGATGGCAATGGAATTA -3′
		**Reverse:** 5′- CATCATGGCCACAAACTCTG-3′
OsCPK30	Os07g44710	**Forward:** 5′- GGGCAACCGACCTATTGTAA -3′
		**Reverse:** 5′- GGCGTGTCTGATCCAATCTT-3′
OsCPK31	AK110341	**Forward:** 5′- GGACGAGCGGATCTCTGA -3′
		**Reverse:** 5′- GTCACGCAGCAGGTTGTAGA-3′
OsCCaMK	AK070533	**Forward:** 5′- AACTCACGAGGCGATGATGCTCTT -3′
		**Reverse:** 5′- GCCTTGAACTCGTCGAAGGTGA -3′
OsSYMRK	AK099778	**Forward:** 5′- TGGCAAGGTTGCGGACTTTGGTTT -3′
		**Reverse:** 5′- TGCATGCTGATGCGACCTCAAGAA -3′
OsPOLLUX	AK072312	**Forward:** 5′- ACAATGGCTTCTGTCACAGCTCCT -3′
		**Reverse:** 5′- TGAACGTATGCACATCTCGTTGCC -3′
OsCASTOR	AK068216	**Forward:** 5′- TGTCTTGGAGGAGCTATTTGCGGA -3′
		**Reverse:** 5′- - TCCGCCTTGAAACTTTGTCTGGTG 3′
OsUbi1	AK121590	**Forward:**5´-CACCTTGGCTGACTACAACATCCA-3´
		**Reverse:** 5'-TGCTTACCAGCAAAGATCAGACGC-3´
OsAct1	AK100267	**Forward:** 5′- CGACGAGTCTGACCCATCCA-3′
		**Reverse:** 5′- GTACCCGCATCAGGCATCTG -3′
