# Supplementary material for: A rice calcium-dependent protein kinase is expressed in cortical root cells during the presymbiotic phase of the arbuscular mycorrhizal symbiosis
Source: BMC Plant Biol. 2011 May 19;11:90. doi: 10.1186/1471-2229-11-90 (PMC3125349; doi:10.1186/1471-2229-11-90)
Supplement: Additional file 9 — Supplementary Methods. Gene expression analysis by semi-quantitative RT-PCR. [file 1471-2229-11-90-S9.PDF]

# Supplementary Methods

## Gene expression analysis by semi-quantitative RT-PCR

The first strand cDNA was synthesized from 1 μg of DNase-treated total RNA using M-MLV reverse transcriptase with 500 ng anchored oligo-(dT) primers in a 20 μl reaction. Gene-specific primers for selected *CPK* genes were designed using the Primer Express software (Applied Biosystems, Norwalk, CT, USA). Primer sets spanning intron regions for each *CPK* gene were designed to confirm that the RT-PCR products were from RNA transcripts rather than from genomic DNA. The expression of rice *SYM* genes was also examined in the same RNA samples used for the analysis of *CPK* gene expression. The rice *SYM* genes studied in this work were: *OsCASTOR* (*Os03g62650*, the rice orthologue of *LjCASTOR* and *MtDIM1a*), *OsPOLLUX* (*Os01g64980*, the rice ortologue of *LjPOLLUX* and *MtDIM1b*), *SYMRK* (*Os07g38070*, the rice ortologue of *LjSYMRK* and *MtDIM2*), and *OsCCaMK* (*Os05g41090*, the rice ortologue of *LjCCaMK* and *MtDIM3*). As controls, the rice *Ubiquitin 1* (*OsUbi1*) and *Actin 1* (*OsAct1*) genes were used. All the primers used in this work are listed in Additional file1: Table S1. Each PCR reaction consisted of 1 μl cDNA in a 10 μl reaction with the following components: 1 μl 10x reaction buffer, 0.2 μl 10 mM dNTP mix, 0.2 μl each primer (10 μM), 0.4 units of Taq DNA polymerase (Invitrogen, Carlsbad, CA, USA). Three independent experiments were performed. For each biological sample, RNA samples were obtained from two independent aliquots of the pulverized plant material, and at least three technical replicates from each RNA sample were analyzed by RT-PCR. Sequence information confirmed that the RT-PCR products obtained were the expected gene fragments.
